# Longitudinal, Non-invasive Live Near-Infrared Fluorescence Imaging of Acute Vascularized Composite Allograft Rejection Validated by *Ex Vivo* ^19^F Magnetic Resonance Imaging and Histopathology

**DOI:** 10.7150/ntno.139245

**Published:** 2026-10-09

**Authors:** Jelena M. Janjic, Yalcin Kulahci, Fatih Zor, Sanjida Ahmed Srishti, Riddhi Vichare, Lesley M. Foley, Zeynep Demir, Bedreddin Sazoğlu, Fatma Nurefsan Selek, Omer Dirican, Vidya Surti, Stefan Stratimirovic, Caitlin Crelli, Rebecca McCallin, Michele Herneisey, Huseyin Karagoz, T. Kevin Hitchens, Vijay S. Gorantla

**Affiliations:** 1 Nanomedicine Manufacturing Laboratory, Graduate School of Pharmaceutical Sciences and School of Pharmacy, Duquesne University, Pittsburgh, PA, USA.; 2 Wake Forest Institute for Regenerative Medicine, Advocate Health - Atrium Wake Forest Baptist Medical Center, Winston-Salem, NC, USA.; 3 Advanced Imaging Center, University of Pittsburgh, Pittsburgh, PA, USA.; 4 Department of Neurobiology, University of Pittsburgh, Pittsburgh, PA, USA.

**Keywords:** near-infrared fluorescence imaging, vascularized composite allotransplantation, acute rejection, cell tracking, fluorine-19 magnetic resonance imaging

## Abstract

Acute rejection remains a major cause of graft attrition in vascularized composite allotransplantation (VCA). Clinical assessment and superficial biopsy may fail to detect early or spatially heterogeneous inflammatory infiltration, particularly within deeper graft compartments. Because macrophages are among the earliest mediators of alloimmune activation, they represent an attractive target for noninvasive immune surveillance of skin-bearing grafts. We evaluated longitudinal near-infrared fluorescence (NIRF) imaging of macrophage infiltration using a perfluoro-15-crown-5 ether nanoemulsion (PCE-NE) labeled with a near-infrared fluorophore in a rat hindlimb transplantation model and a radial forearm flap model in non-human primates (NHPs). Single- and dual-dose administration strategies enabled serial visualization of macrophage recruitment and improved detection of evolving inflammatory responses during acute rejection. To capture the spatial heterogeneity of rejection, a multi-sampled region-of-interest analysis framework was developed for quantitative mapping of NIRF signal across donor and recipient tissues in rats and throughout transplanted flap regions in NHPs. To validate *in vivo* imaging findings, grafts were analyzed *ex vivo* using fluorine-19 (^19^F) magnetic resonance imaging, histopathology, and CD68 immunofluorescence. NIRF signal identified rejecting graft regions and demonstrated spatial patterns of inflammatory infiltration that corresponded to compartment-specific ^19^F MRI signal localization in *ex vivo* imaging. Imaging findings corresponded with Banff rejection grade and CD68-positive macrophage accumulation across Grade I-IV rejection. Importantly, increased NIRF and ^19^F MRI signals were detected during early Grade I-II rejection, when clinical manifestations remained limited. These findings establish a translational multimodal imaging strategy for longitudinal, noninvasive monitoring of acute VCA rejection and demonstrate concordance between *in vivo* NIRF imaging, *ex vivo* ^19^F MRI, histopathology, and immunofluorescence across rodent and non-human primate models.

## 1. Introduction

Vascularized composite allotransplantation (VCA) has transformed reconstructive transplantation; however, acute rejection (AR) remains a frequent and clinically significant barrier to durable graft survival and long-term functional outcomes^1^. Rejection episodes are often recurrent and contribute to cumulative tissue injury, underscoring the need for improved strategies for longitudinal graft surveillance beyond episodic clinical assessment and biopsy^1^, ^2^. Current monitoring relies primarily on visual examination and serial skin biopsy. While accessible, these approaches are limited in their ability to provide continuous, noninvasive longitudinal tracking of evolving immune activity. Importantly, early Banff Grade I-II rejection may present with only subtle erythema or edema, making clinical delineation unreliable and delaying therapeutic intervention^3^, ^4^. A platform capable of detecting immune activation prior to overt clinical deterioration would therefore enable earlier and potentially preemptive rejection management.

Macrophages are among the earliest immune cells recruited during alloimmune activation and play a central role in coordinating adaptive immune responses through cytokine signaling and interaction with T and B lymphocytes^5^, ^6^. Their early infiltration into graft tissue makes them an attractive mechanistic biomarker for immune activation. Imaging macrophage burden longitudinally and non-invasively could therefore provide a clinically translational approach for the detection of acute rejection in real time^6^. Here, we report a multimodal imaging strategy to monitor macrophage-driven acute rejection in vascularized composite allotransplantation (VCA), integrating near-infrared fluorescence (NIRF) with *ex vivo* fluorine-19 magnetic resonance imaging (^19^F MRI) and macrophage detection in graft tissues.

NIRF imaging offers sensitive, real-time detection of labeled macrophages within superficial tissues^7^-^9^ and is compatible with rapid bedside and intraoperative assessment. However, it is inherently depth-limited and two-dimensional. Together, these modalities offer complementary strengths: NIRF for high-throughput, longitudinal, non-invasive monitoring over time and ^19^F MRI for quantitative compartment-level confirmation *ex vivo*^10^. Skin-bearing vascularized composite allotransplants are uniquely suited to such imaging strategies because they are externally visible grafts amenable to optical imaging while also containing substantial soft tissue accessible to MRI.

In this study, we developed a macrophage-specific perfluoro-15-crown-5 ether nanoemulsion (PCE-NE) as a multimodal nanoimaging probe for sequential near-infrared fluorescence and fluorine magnetic resonance imaging in a rat hindlimb transplant model 11. To address the spatially heterogeneous distribution of inflammation across graft compartments, we implemented longitudinal quantitative NIRF imaging coupled with a multi-sampled region-of-interest (ROI) analysis strategy to enable spatially resolved assessment of macrophage-associated signal over time.

To facilitate clinical translation, we further evaluated this platform in a pilot cohort of nonhuman primates undergoing fasciocutaneous radial forearm flap (RFF) transplantation using a clinical-grade PCE-NE formulation and a clinically approved FLUOBEAM imaging system. This large-animal study was designed to assess the feasibility, persistence, and quantification of macrophage-associated NIRF signal within a clinically relevant VCA model.

To our knowledge, this is the first study in VCA to integrate noninvasive longitudinal macrophage tracking with near-infrared fluorescence imaging in living subjects, quantitative ^19^F MRI for compartment-level spatial mapping *ex vivo*, immunohistochemistry and immunofluorescence imaging, and histological characterization of acute rejection across Banff grades. Furthermore, we demonstrate the translational feasibility of this imaging strategy in a clinically relevant nonhuman primate model. Together, these studies establish a multimodal framework for sequential immune surveillance and depth-resolved confirmation of inflammatory distribution within vascularized composite allografts.

## 2. Methods

### 2.1. Rat Study design overview

Fully mismatched Brown Norway→Lewis orthotopic hindlimb transplants were performed to model acute rejection through postoperative day (POD) 6-8 (Banff Grade I-IV). Nanoemulsions were administered either as a single intravenous dose on POD0, as dual doses on POD3 and POD6, or as three doses on POD0, POD3, and POD6 to enhance labeling of rejection-associated macrophages. Longitudinal *in vivo* near-infrared fluorescence (NIRF) imaging was performed until endpoint, followed by *ex vivo* NIRF, ^19^F/^1^H magnetic resonance imaging (MRI), and histological assessments that correspond to the presence of macrophages to *ex vivo* ^19^F MR and NIRF signal. All animal experiments were performed in accordance with institutional and national guidelines for the care and use of laboratory animals and were approved by the Institutional Animal Care and Use Committee (IACUC) of Wake Forest Institute for Regenerative Medicine, Atrium Health, Winston-Salem, NC.

### 2.2. NHP Study design overview

Longitudinal *in vivo* near-infrared fluorescence (NIRF) imaging was performed until endpoint, followed by *ex vivo* NIRF and histologic correlation. All animal experiments were performed in accordance with institutional and national guidelines for the care and use of laboratory animals and were approved by the Institutional Animal Care and Use Committee (IACUC) of Wake Forest Institute for Regenerative Medicine, Atrium Health, Winston-Salem, NC.

### 2.3. Rat hindlimb transplant model

Orthotopic hindlimb transplantation was performed using established microsurgical techniques. The model incorporates skin, muscle, nerve, and vascular compartments to recapitulate multi-compartment rejection (Figure S1). No maintenance immunosuppression was administered. Animals were monitored daily and euthanized at POD7-8 or at humane endpoints.

### 2.4. Non-human primate radial forearm fasciocutaneous (RFF) model

A pilot cohort of two adult cynomolgus macaques (Macaca fascicularis; 2.5-4.0 kg, 2-4 years old) was utilized as a large-animal translational model of vascularized composite allotransplantation (VCA). All animals were obtained from accredited vendors, confirmed pathogen-free, and housed in AAALAC-accredited facilities. Experimental procedures were approved by the Institutional Animal Care and Use Committee (IACUC) of Wake Forest Institute for Regenerative Medicine, Atrium Health, and the U.S. Army Animal Care and Use Review Office (ACURO) and were conducted in accordance with the Animal Welfare Act, the National Institutes of Health Guide for the Care and Use of Laboratory Animals, and Department of Defense Directive 3216.01. Animals underwent radial forearm fasciocutaneous (RFF) flap allotransplantation under general anesthesia. Donor flaps consisted of skin, subcutaneous tissue, brachioradialis muscle, palmaris longus tendon, medial and lateral antebrachial cutaneous nerves, and the radial artery and cephalic vein vascular pedicle. Following flap procurement and cold preservation, recipient transplantation was performed using microsurgical end-to-end arterial and venous anastomoses and neurorrhaphy of the corresponding sensory nerves. Following restoration of graft perfusion, recipients received a single dose of intravenous administration of PCE-NE (1 mL/kg) through a central venous catheter on postoperative day (POD) 0. Longitudinal near-infrared fluorescence (NIRF) imaging was performed using a clinical-grade FLUOBEAM imaging system to evaluate macrophage-associated signal persistence and rejection-associated inflammatory changes over time. Quantitative image analysis was performed using the same region-of-interest (ROI) methodology applied in the rodent hindlimb transplantation studies to enable cross-species comparison of imaging signal dynamics. Acute rejection progressed to graft failure in both animals, resulting in study endpoints on POD8 and POD7 (NHP A and NHP B, respectively). At study termination, graft tissues were collected for *ex vivo* imaging, histological evaluation, and immunohistochemical analysis.

### 2.5. Multimodal nanoimaging probe preparation and administration

A nanoimaging probe, formulated as a Perfluoro-15-crown-5 ether (PCE) nanoemulsion incorporating a near-infrared fluorophore, was manufactured by high-pressure microfluidization as previously described^11^. Droplet size and fluorine content were confirmed by dynamic light scattering and ^19^F NMR^11^. In rats, nanoemulsions (0.5 mL IV) were administered via the external jugular vein according to single- or dual-dose protocols.

### 2.6. Near-infrared fluorescence imaging and signal quantification

#### 2.6.1. Rat NIRF imaging analysis: *in vivo* and *ex vivo*

*In vivo* and *ex vivo* near-infrared fluorescence (NIRF) imaging was performed using LI-COR Pearl^®^ Small Animal Imager (LI-COR Biosciences, Lincoln, NE, USA) in rat hindlimbs with optimized settings in Image Studio™ Software (Version 5.2, LI-COR Biosciences, Lincoln, NE, USA). Live NIRF images were captured at 800 nm channel using 85 µm or 170 µm resolution settings. In the *ex vivo* imaging both orientations of the allograft, including the inner side (View 1) and outer side (View 2), were imaged and accounted for in fluorescence signal quantification at 800 nm channel at a resolution of 85 µm and focus offset of 2 settings. To quantify the fluorescence signal, a multi-sampled region of interest (ROI) strategy was utilized across the transplanted rat hindlimb. The distinct regions of the transplanted hindlimb were sampled in multiple ROIs and the fluorescence signal for each region in the hindlimb was quantified using Image Studio (Version 5.2). Based on the biological view of transplanted limbs, multiple ROIs were captured and the same area for each circled region was maintained to standardize measurements in experimental groups. The background signal was normalized, and NIRF images were linked to compare fluorescence signal intensity across all groups. Using the same approach, multiple ROIs were also selected across the entire limb, including the transplanted hindlimb and adjacent recipient tissue, to characterize the spatial distribution of the fluorescence signal, which corresponds to the PCE-NE-labeled monocyte-derived macrophages. Fluorescence signals were analyzed by performing one-way analysis of variance (ANOVA) to determine statistical significance among postoperative time points and experimental groups.

#### 2.6.2. NHP *in vivo* and *ex vivo* imaging analysis

Longitudinal *in vivo* and *ex vivo* near-infrared fluorescence (NIRF) imaging was performed using the FLUOBEAM^®^ LX imaging system (Getinge AB, Göteborg, Sweden) to monitor flap transplantation and rejection. Images were acquired using standardized settings (80 ms exposure, ×10 resolution). *Ex vivo* image analysis was performed using Image Studio™ Software (v5.2, LI-COR Biosciences, Lincoln, NE, USA), and tissues were imaged in both skin-up and skin-down orientations to evaluate fluorescence distribution and dye penetration. Fluorescence signal was quantified from the green channel using Image Studio™ Software. Two ROI-based approaches were used to assess macrophage-associated fluorescence dynamics. Method 1 employed identically sized circular ROIs arranged within flap boundaries to quantify fluorescence localized to the transplanted tissue. Method 2 employed identically sized circular ROIs distributed across the flap and adjacent limb tissue to assess regional fluorescence changes. ROI size and number were maintained across imaging sessions. For NHP A, ROIs overlapping fluorescent surgical tape on postoperative days 1-2 were excluded from analysis. Fluorescence values were background-corrected, normalized to ROI area, and compared across imaging time points using linked image analysis. Following tissue harvest, excised flap specimens were imaged using the same acquisition settings as *in vivo* imaging. Fluorescence measurements were background-corrected and normalized to a low-signal reference region. Regions exhibiting weak and strong fluorescence signals were identified, and triplicate circular ROIs were used for signal quantification. Mean fluorescence intensity for each region was calculated from triplicate measurements. Statistical significance between weak- and strong-signal regions was assessed using an unpaired two-tailed t-test.

### 2.7. Fluorine MRI acquisition and quantification

*Ex vivo* MRI was performed at 9.4 Tesla using a dual-tuned ^19^F/^1^H resonator. Co-registered anatomical and fluorine images were acquired, and a PCE reference standard was used for quantification. Regions of interest were delineated by anatomical compartment, and fluorine concentration was calculated relative to the standard.

### 2.8. Macrophage Quantification from Stained Tissue Images

Tissue samples from NHPs were fixed in 10% neutral buffered formalin for 24 h and processed by HistoWiz Inc. (Brooklyn, NY, USA) for paraffin embedding, sectioning, CD68 immunohistochemical staining, and whole-slide scanning. Sections (4 μm) were analyzed in QuPath (v.0.5.1). Images were configured as H-DAB, stain vectors were estimated automatically, and tissue regions were manually annotated. Cell detection was performed using QuPath default watershed-based parameters. A Random Trees classifier was trained using manually annotated macrophage and non-macrophage populations based on nuclear, cytoplasmic, and whole-cell DAB optical density features. Macrophage percentage was calculated as CD68-positive cells divided by total cell count for each section. Weighted averages and standard deviations were calculated using total cell counts per section. Statistical comparisons between weak- and strong-signal regions were performed using an unpaired two-tailed t-test.

### 2.9. Histological Assessment

Full-thickness graft tissue was collected at endpoint for hematoxylin and eosin staining and Banff grading. CD68 immunohistochemistry and immunofluorescence were performed to confirm macrophage localization and imaging-histology concordance.

### 2.10. Statistical Analysis

Data are presented as mean ± standard deviation. One-way ANOVA or Student's t-test was used as appropriate. A p-value < 0.05 was considered significant. All statistical analyses were performed on GraphPad Prism 10.0.

## 3. Results

### 3.1. Development and *in vitro* optimization of a macrophage-specific nanoimaging probe for rejection imaging

Given the extensive tissue inflammation characteristic of acute rejection in VCA, the formulation incorporated increased levels of both PCE and NIRF dye, and the dosing strategy was adapted to capture multiple waves of infiltrating monocyte-derived macrophages. We have previously demonstrated successful NIRF tracking of macrophage infiltration in rodent models of inflammation, injury, and trauma following single or multiple intravenous doses of PCE-NE^7^, ^8^, ^12^-^17^. Here, we prepared a multimodal nanoimaging probe, customized for VCA acute rejection, as a triphasic nanoemulsion (PCE-NE) comprising a perfluoro-15-crown-5 ether (PCE) core, a triglyceride phase, and non-ionic surfactants, with a low droplet diameter (~120 nm). **Figure 1** summarizes the structural and functional characteristics of this VCA-tailored nanoimaging probe. The characteristic ^19^F NMR spectrum confirms the integrity of the fluorine payload and its suitability for quantitative MR tracking (**Figure 1A**). Droplet size distribution was maintained within the optimal range shown in **Figure 1B** for *in situ* labeling of monocyte-derived macrophages following intravenous administration in rats^7^, ^12^, ^13^. Importantly, the PCE-NE demonstrated stability under physiologically relevant, serum-containing conditions, supporting translational robustness after systemic delivery (**Figure 1C**). Reliable longitudinal detection requires a stable optical signal; accordingly, fluorescence from the embedded DiR dye was preserved across dilutions, enabling consistent *in vivo* and *ex vivo* NIRF imaging (**Figure 1D**). *In vitro* evaluation further supported biologic compatibility. Macrophage viability was preserved across the tested dose range, and fluorescence microscopy confirmed efficient intracellular uptake, consistent with suitability for tracking macrophage infiltration in VCA acute rejection (**Figure 1E & 1F**). Collectively, these data validate the VCA-customized PCE-NE as a stable, biocompatible, and translationally viable multimodal probe capable of supporting longitudinal NIRF detection and *ex vivo* quantitative ^19^F MRI tracking of macrophage infiltration in acute rejection.

### 3.2. Sequential NIRF imaging with a single-dose PCE-NE after VCA

To establish baseline *in vivo* kinetics of macrophage-associated signal during evolving rejection, a single intravenous dose of PCE-NE was administered immediately after transplantation (POD0), followed by serial NIRF imaging through POD6 (**Figure 2A-B**). Clinically, grafts progressed from minimal early postoperative changes to increasing erythema, edema, desquamation, and tissue injury, consistent with escalating Banff grades^1^. Histopathologic evaluation confirmed stepwise progression from Banff Grade 0 in the early postoperative period to Grade I by POD3 and advanced Grade III-IV rejection by POD5-6 **(Figure 2C).** In parallel, NIRF imaging demonstrated early focal fluorescence within the transplanted limb beginning at POD2, preceding overt high-grade clinical deterioration. Early postoperative signal with a single POD0 dose of PCE-NE likely reflects a combination of ischemia-reperfusion-associated inflammation and emerging alloimmune recruitment. These baseline kinetics established the temporal relationship between the PCE-NE signal and Banff progression and provided the biological rationale for repeated dosing strategies to more comprehensively map macrophage dynamics and distribution during acute rejection.

### 3.3. Macrophages localize to adnexal niches and deep dermal interface during Banff Grade III rejection

In Banff Grade III rejection, the inflammatory burden extended well beyond superficial dermal involvement, forming characteristic deep dermal interface bands and dermal-subcutaneous transition zones. Histopathologic evaluation (**Figure 3**) showed dense mononuclear aggregates tracking along perivascular structures and concentrating at the dermal interface, architectural features consistent with advanced alloimmune injury rather than nonspecific postoperative edema. Prominent macrophage enrichment was also observed surrounding adnexal and follicular units, highlighting the compartmental complexity of late-stage rejection^18^. Immunofluorescence analysis revealed tight spatial concordance between CD68⁺ macrophages and PCE-NE fluorescence within these deep interface, perivascular, and perifollicular regions. Areas devoid of CD68⁺ cells showed minimal probe-associated signal, suggesting that nanoemulsion uptake was predominantly macrophage-dependent rather than driven by passive diffusion or vascular leak. The embedded lipophilic fluorophore within the PCE-NE droplet enabled direct histologic visualization of labeled macrophages, providing cellular-level validation of the *in vivo* NIRF findings and complementing subsequent ^19^F MRI-based detection. Collectively, these data confirm that PCE-NE fluorescence corresponds to the tissue distribution of macrophages in advanced acute rejection. The probe therefore functions as a biologically specific, multimodal imaging agent that reports macrophage infiltration across dermal, interface, vascular, and adnexal compartments.

### 3.4. Alloimmune macrophage recruitment resolved through novel spatially stratified live NIRF image sampling

Acute rejection in the rat hindlimb VCA model exhibits spatial heterogeneity across superficial and deeper tissue compartments. Initial quantification of NIRF signal across broad anatomical regions—the transplanted limb, recipient stump, and entire post-transplant limb (**Figure S2**)—revealed non-uniform signal distribution that was not adequately captured by single-region measurements. To address this spatial complexity, we developed a multi-sampled region-of-interest (ROI) image analysis strategy (**Figure 4B-D**) designed to resolve intra-limb heterogeneity and map signal gradients across overlapping donor and recipient compartments. As an initial methodological evaluation, we applied a multi-sampled ROI approach to a transplanted hindlimb (one donor-recipient pair) to characterize within-graft spatial heterogeneity before evaluating variability across animals with differing rejection trajectories. Therefore, n denotes the number of ROIs representing spatial subsamples of the same graft, not independent biological replicates (N = 1 transplant recipient). This approach enabled quantitative assessment of the NIRF signal distribution with improved spatial resolution and statistical power, thereby increasing sensitivity to detecting temporal changes in macrophage-associated signal during rejection progression. This approach allowed us to more accurately assess differences in macrophage infiltration patterns across the transplanted region at different time points post-transplantation.

POD0 dose captures early post-surgical inflammatory events associated with ischemia-reperfusion injury (IRI), as shown in a single-dose experiment (**Figure 2**). Since this phase also has neutrophil-dominant inflammation^19^, ^20^, we used POD3 and POD6 administrations without POD0 dosing to label sequential waves of monocyte-derived macrophage recruitment associated with evolving alloimmune rejection. Serial imaging demonstrated progressive emergence beginning at POD4 and continued spatial expansion through POD6-7 (**Figure 4**). This delayed amplification is consistent with labeling of newly recruited monocyte-derived macrophages rather than persistence of early ischemia-reperfusion-associated inflammatory cells. The emergence of the signal approximately 24 h post-injection at POD3 is consistent with earlier findings in rat models of solid-organ rejection^10^. The temporal separation between early postoperative inflammation and later signal escalation suggests the NIRF signal corresponds to rejection-associated macrophage recruitment.

When fluorescence was quantified across the full imaged hindlimb field, signal increased progressively over time, reflecting cumulative inflammatory burden across overlapping donor and proximal recipient compartments. However, multi-sampling ROI restriction to the distal donor graft revealed a distinct infiltration pattern: a quantitatively modest signal through POD5, followed by a significant escalation at POD6-POD7 (**Figure 4D**). This divergence suggests preferential macrophage enrichment within rejecting donor tissue as alloimmune activation intensifies. Spatial mapping further demonstrated expansion from focal signal regions to broader dermal and limb-wide coverage at later time points, consistent with staged inflammatory amplification (**Figure 4C**). Together, these findings show that repeated PCE-NE administration enhances the temporal resolution of macrophage kinetics and improves the discrimination of rejection-associated immune recruitment from early surgical inflammation, providing the mechanistic basis for the subsequent implementation of a three-dose regimen.

### 3.5. Three-dose regimen of PCE-NE enhances *in vivo* mapping of macrophage recruitment

To distinguish early postoperative inflammation from subsequent alloimmune-driven macrophage recruitment, we implemented a three-dose PCE-NE regimen (POD0, POD3, POD6), **Figure 5A**, designed to capture temporally distinct waves of macrophage infiltration. Serial NIRF live imaging demonstrated progressive signal amplification across the graft, with maximal fluorescence following the POD6 administration (**Figure 5B & 5C**). Importantly, signal escalation at later time points occurred despite resolution of immediate surgical inflammation, supporting accumulation of newly recruited macrophage populations associated with active rejection rather than residual innate injury. This stepwise amplification is consistent with continued macrophage-associated probe accumulation during evolving rejection; however, the present study cannot distinguish the relative contributions of newly labeled macrophages and the persistence of PCE-NE from earlier administrations. *Ex vivo* imaging confirmed robust graft-localized fluorescence in rejecting allografts compared with minimal signal in non-rejecting controls (**Figure 5D**). Spatial heterogeneity within the graft reflected the localization of PCE-NE to zones of rejection, further confirmed with the ^19^F NMR spectra (**Figure S4**). ROI-based multisampling quantification including both the inner and outer views of the transplanted limb demonstrated significantly (p < 0.0001) elevated PCE-NE-associated NIRF signal in rejecting grafts (**Figure 5E**), concordant with marked increases in CD68⁺ macrophage density**.** Quantification of CD68⁺ macrophages (**Figure S5**) in representative immunohistochemistry samples demonstrated that the *in vivo* NIRF signal corresponded to dense macrophage infiltration within rejecting tissue compartments, supporting the biological specificity of PCE-NE uptake (**Figure 5F & 5G**). Together, these data demonstrate that repeated PCE-NE administration enhances the sensitivity of detecting evolving macrophage recruitment and enables discrimination and monitoring rejection-associated immune amplification and early post-surgical inflammation. By capturing sequential waves of macrophage recruitment, the three-dose regimen enables dynamic immune surveillance across the rejection trajectory, strengthening the translational potential of PCE-NE-based NIRF imaging as a non-invasive strategy for monitoring rejection in vascularized composite allografts.

### 3.6. NIRF imaging of PCE-NE-labeled macrophages during early Banff Grade I-II rejection

To determine whether temporally optimized macrophage labeling improves early rejection detection, we evaluated grafts using the three-dose PCE-NE regimen (POD0, POD3, POD6), designed to capture both the initial inflammatory response and subsequent alloimmune-driven macrophage waves (**Figure 6**). At Banff Grade I (POD3), macrophage infiltration was already evident within deep dermal and perivascular compartments when visible clinical changes were limited to mild erythema or edema, and clinical delineation was less reliable. *In vivo* NIRF imaging demonstrated a clear graft-localized signal at this stage, indicating that repeated labeling captures early immune activation that may be underestimated by visual inspection alone. This supports the concept that the secondary macrophage wave emerging around POD3 is effectively detected with repeated dosing. With progression to Banff Grade II, inflammation expanded into deeper dermal and vascular regions. NIRF signal remained robust within the graft and correlated with histologic evidence of macrophage accumulation. Immunohistochemical and immunofluorescent analyses confirmed that fluorescence localized specifically to CD68⁺ macrophages, including perivascular aggregates surrounding dermal arterioles, validating cellular uptake-dependent signal generation rather than nonspecific enhancement. These findings demonstrate that the three-dose PCE-NE regimen enables sensitive, macrophage-specific detection of early rejection stages and improves immune surveillance during the critical transition from innate postoperative inflammation to alloimmune amplification. NIRF signal remained robust within the graft and corresponded with histologic evidence of macrophage accumulation. These findings demonstrate that macrophage-associated NIRF signal can be detected during histologically confirmed early Banff Grade I-II rejection when gross clinical manifestations remain limited. By identifying macrophage recruitment at Banff Grade I-II before advanced tissue injury develops, this approach offers a non-invasive strategy that could facilitate earlier therapeutic intervention and reduce delayed or missed diagnosis in vascularized composite allotransplantation.

### 3.7. Whole-limb ^19^F MRI confirms spatial macrophage infiltration in deep tissues during acute rejection

We performed whole-limb *ex vivo* ^19^F MRI to spatially map PCE-NE-labeled macrophages across deeper graft compartments to determine whether macrophage-associated signal extended beyond superficial imaging with the nanoimaging probe. Compartment-specific quantification confirmed significantly greater fluorine signal burden across multiple graft tissues, demonstrating that macrophage infiltration during acute rejection is anatomically extensive and not confined to superficial skin compartments (**Figure 7**). In rejecting allografts, fluorine signal was distributed throughout the full thickness of the limb, including deep muscle groups, fascial planes, foot musculature, and marrow-adjacent regions. Signal was particularly enriched at the surgical interface, consistent with a focal amplification zone of inflammatory recruitment. In contrast, contralateral control limbs demonstrated minimal fluorine signal, largely restricted to physiologic bone marrow compartments. Notably, signal within deep muscle and fascial planes highlights inflammatory involvement that may be under-sampled by standard skin biopsies. These data establish that PCE-NE-labeled macrophages distribute throughout the rejecting graft and that ^19^F MRI provides depth-resolved validation of nanoemulsion-based immune imaging beyond the surface sensitivity of NIRF.

### 3.8. PCE-NE enables multimodal monitoring of acute rejection in superficial and deep compartments of VCA

To evaluate whether macrophage-targeted PCE-NE imaging could unify surface findings with depth-resolved inflammatory architecture, we performed combined NIRF and ¹H/^19^F MRI across early and advanced rejection stages. In **Figure 8**, at POD3 (Banff Grade I-II), when clinical findings remained subtle, NIRF detected clear macrophage-associated signal within the graft, indicating early immune activity not fully appreciated on gross examination. With progression to Banff Grade III rejection, signal intensity increased and expanded spatially, reflecting escalating inflammatory burden. Depth-resolved ^19^F MRI demonstrated that macrophage accumulation was not confined to superficial compartments but extended across fascia, muscle groups, and peri-surgical regions, establishing graft-wide involvement. In contrast, contralateral limbs showed minimal fluorine signal, largely restricted to physiologic marrow compartments. Histopathologic evaluation confirmed dense mononuclear infiltration in MRI-positive regions, and immunofluorescence demonstrated concordant PCE-NE localization within CD68⁺ macrophages. The absence of diffuse signal in CD68-negative areas supports a cellular uptake-mediated mechanism rather than nonspecific interstitial accumulation. Collectively, these data establish that the optimized three-dose PCE-NE regimen enables spatially resolved, biologically validated mapping of macrophage infiltration across tissue compartments. Surface-sensitive NIRF detection and depth-penetrant ^19^F MRI provide complementary readouts of rejection activity, supporting a clinically translatable framework for non-invasive immune surveillance in vascularized composite allotransplantation.

### 3.9. Translation of Macrophage-Targeted Nanoemulsion Imaging Paradigm to a Large-Animal NHP Radial Forearm Flap VCA Model

To evaluate the translational feasibility of longitudinal macrophage-associated NIRF imaging demonstrated in the rat VCA model, the platform was extended to a pilot cohort of two nonhuman primates (NHPs) undergoing fasciocutaneous radial forearm flap (RFF) transplantation. This cohort served as a proof-of-concept large-animal validation using a clinical-grade nanoemulsion (NE) formulation and the clinical-grade FLUOBEAM^®^ imaging system. Animals received a single intravenous NE dose (1 mL/kg) on postoperative day (POD) 0, allowing assessment of the persistence and utility of a one-time administration strategy during acute rejection. Notably, fluorescence signal remained detectable for up to 7-8 days following administration, spanning the entire rejection course in both animals (**Figure S6**). The two NHPs developed acute rejection requiring flap explantation on POD8 and POD7 (NHP A and NHP B, respectively). To enable direct comparison with the rat studies, fluorescence signal quantification was performed using the same region-of-interest (ROI) methodology, including both flap-restricted and expanded analyses, thereby allowing evaluation of macrophage localization and signal distribution across species.

For NHP A, analysis of the restricted flap ROI (**Figure 9A**, first column) demonstrated significantly increased fluorescence signal on postoperative day (POD) 1 (p < 0.0001), POD2 (p < 0.0001), POD3 (p < 0.0001), POD5 (p < 0.001), and POD8 (p < 0.01). This kinetic profile was consistent with acute rejection, characterized by a pronounced early increase in signal intensity at POD1 followed by a gradual decline that remained elevated through the onset of clinical rejection at POD8. In contrast, analysis using the expanded ROI (**Figure 9A**, second column) revealed significantly decreased signal intensity on POD2 (p < 0.01), POD3 (p < 0.001), POD5 (p < 0.0001), and POD8 (p < 0.0001). Rather than demonstrating localized signal accumulation within the flap, this approach produced an overall declining trend from baseline, interrupted only by a transient increase at POD4.

A similar pattern was observed in NHP B. Analysis of the restricted flap ROI (**Figure 9A**, third column) showed significantly elevated fluorescence signal on POD1 (p < 0.01), POD2 (p < 0.01), POD3 (p < 0.0001), POD4 (p < 0.0001), POD5 (p < 0.001), and POD7 (p < 0.05). As observed in NHP A, signal intensity peaked early and subsequently declined over time while remaining elevated relative to baseline. However, the expanded ROI analysis (Figure 9A, fourth column) demonstrated a distinct kinetic profile, with significantly increased signal on POD1 (p < 0.0001), POD3 (p < 0.0001), POD4 (p < 0.01), and POD5 (p < 0.01), followed by a marked decrease on POD7 (p < 0.0001). This profile was characterized by sustained signal elevation preceding an abrupt decline at the time of rejection. Collectively, these findings demonstrate that fluorescence measurements confined to the transplanted flap more accurately capture localized macrophage accumulation associated with rejection than analyses incorporating surrounding host tissue.

To define the clinical extent of rejection in this NHP VCA model, serial photographic assessment demonstrated progressive graft deterioration characterized by dermal erythema, edema, and epidermolysis localized primarily to the central vascular territory of the flap (**Figure 9B**). To further characterize macrophage distribution and NE uptake, dual-immunofluorescence staining was performed on rejecting flap tissues. Merged DAPI, CD68, and NE fluorescence images revealed substantial macrophage accumulation and NE localization within perivascular and deep dermal compartments (**Figure 9B**, bottom row). Histologic evaluation by H&E staining demonstrated prominent perivascular and periadventitial inflammatory cuffing, consistent with perivasculitis and deep dermal rejection (**Figure 9B**, top right).

To validate the relationship between fluorescence signal intensity and macrophage infiltration, *ex vivo* fluorescence imaging was performed on the NHP A flap following explantation (**Figure 9C**). Regions exhibiting visually strong and weak fluorescence signals were identified on FLUOBEAM images (Figure 9C, top middle). Quantitative Image Studio analysis confirmed that the high-signal region exhibited significantly greater fluorescence intensity than the low-signal region (p < 0.0001) (**Figure 9C**, top right). Tissue samples were subsequently collected from both regions for histologic analysis (**Figure 9C**, bottom left). CD68 immunostaining demonstrated significantly greater macrophage infiltration within tissue obtained from the high-fluorescence region compared with the low-fluorescence region (p < 0.01) (**Figure 9C**, bottom middle and right). These findings establish a direct correlation between non-invasive fluorescence imaging and histologically confirmed macrophage accumulation, supporting the utility of fluorescence-based macrophage tracking as a surrogate biomarker of immune cell infiltration during VCA rejection. These studies establish the feasibility of longitudinal macrophage imaging in a clinically relevant large-animal model and provide a foundation for future investigations evaluating repeated dosing strategies and the utility of this platform for monitoring chronic rejection.

## 4. Discussion

In this study, we demonstrate that a multimodal perfluoro-15-crown-5 ether nanoemulsion (PCE-NE) enables longitudinal, spatially resolved mapping of macrophage infiltration during acute VCA rejection. To the best of our knowledge, this is the first study to demonstrate noninvasive imaging of macrophage infiltration as a biomarker of acute rejection in VCA across both rodent and non-human primate models.

Using Banff-graded histopathology as the reference standard for AR^1^, ^21^, we show that macrophage-associated NIRF signal can be detected at early Banff Grade I-II stages when clinical skin findings are subtle^22^, and that integration of quantitative surface NIRF imaging with depth-resolved ^19^F MRI reveals graft-wide macrophage distribution extending beyond superficial dermis into fascia, muscle, perivascular, and adnexal compartments^18^. Repeated dosing (POD0, POD3, POD6) more comprehensively captured temporally distinct recruitment waves than single-dose labeling, consistent with dynamic innate-to-alloimmune transitions in the post-transplant inflammatory trajectory^2^, ^23^. Immunohistochemistry and immunofluorescence demonstrated spatial concordance between PCE-NE-associated NIRF signal and CD68⁺ macrophage infiltration across rejection stages, supporting macrophage-dependent uptake rather than nonspecific edema or vascular leak.

A key design choice was to optimize and validate the platform first in AR, where standardized grading exists^1^, ^21^ providing a rigorous biological anchor before considering extension to phenotypes such as chronic rejection (CR), for which validated grading remains limited or evolving^24^, ^25^.

Macrophages are increasingly recognized as central mediators of allograft injury across transplant settings, contributing to antigen presentation, inflammatory amplification, endothelial activation, and tissue remodeling^26^. In VCA, clinical and experimental observations support an association between macrophage burden and severe rejection, including reports implicating macrophage-associated mediators such as MMP3 as candidate biomarkers in clinical face transplantation^27^. However, macrophage assessment in current VCA practice remains biopsy-dependent and therefore susceptible to spatial heterogeneity, sampling bias, and limited feasibility for frequent longitudinal surveillance^28^.

^19^F MRI is a well-established translational methodology for background-free cell tracking and quantitative mapping of immune cells in cardiovascular diseases^29^, ^30^, cancer^31^, ^32^, and organ rejection^10^, ^33^. In parallel, nanoemulsion platforms that incorporate both ^19^F tracers (perfluorocarbons) and fluorophores have enabled dual-modality optical/MR readouts in inflammatory settings^9^, ^34^. For the first time, our study extends these principles to VCA AR by combining (i) rapid, longitudinal NIRF detection (bedside-compatible) with (ii) confirmatory ^19^F MRI for depth-resolved, compartment-specific localization (**Figure S3**), and (iii) direct tissue-level visualization of probe signal on immunofluorescence sections via an embedded lipophilic fluorophore**.** This multimodal approach, which integrates clinical exam → NIRF → MRI → histology/IF, provides a biologically validated framework for macrophage-directed rejection imaging anchored to Banff criteria^4^, ^21^, ^23^.

The temporal kinetics observed are consistent with staged post-transplant inflammation in which early IRI and surgical injury initiate innate responses that are often neutrophil-rich, followed by recruitment and expansion of monocyte-derived macrophages during evolving alloimmune activation^20^. In this context, early macrophage-associated signal after POD0 dosing likely reflects a composite of injury/IRI-associated inflammation with emerging alloimmune recruitment^19^. Signal escalation beginning around POD3 aligns with the timing of Banff Grade I-II rejection and is consistent with increasing macrophage accumulation within perivascular and deep dermal compartments typical of progressive AR^35^.

The plateau or decline of signal at later high-grade stages after single-dose labeling supports the concept that a single labeling event may incompletely represent later recruitment waves, particularly if initially labeled macrophage populations turn over and are replaced by newly recruited cells not exposed to the original dose^36^. Repeated dosing therefore provides a practical strategy for capturing temporally distinct macrophage waves and enabling broader full-thickness spatial mapping. Fluorescence during evolving rejection was higher than the early postoperative signal, consistent with additional macrophage-associated probe accumulation. However, initially labeled macrophage populations may also contribute to the signal during the later rejection phase. While our colocalization data support intracellular uptake within CD68⁺ macrophages, the relative contributions of resident versus recruited macrophage subsets and the extent to which uptake varies by activation phenotype remain incompletely defined. Future studies will be required to dissect subset-specific kinetics and to determine whether signal intensity scales linearly with macrophage burden across phenotypic states.

Noninvasive immune surveillance remains an unmet need in VCA, where clinical monitoring is skin-centric and biopsy-based^4^, ^23^, ^37^-^39^. The present data suggest a clinically plausible monitoring hierarchy in which NIRF provides early, bedside sensitivity and ^19^F MRI provides depth-resolved confirmation and compartmental mapping, with Banff-graded histology serving as the reference standard during platform optimization.

The platform also enables orthogonal tissue-specific immunological confirmation. Because the PCE-NE includes an embedded fluorophore, nanoemulsion uptake can be visualized directly on immunofluorescence sections without relying solely on secondary probe-specific antibody detection, strengthening the interpretability of imaging-histology concordance. In addition, macrophage-linked blood biomarkers may provide complementary confirmation. For example, MMP3 has been implicated as a biomarker associated with severe rejection in clinical VCA^27^, ^28^, ^40^, raising the possibility that elevated NIRF signal might be paired with serum biomarker assessment to strengthen risk stratification before escalating to MRI or biopsy.

To further evaluate the clinical translatability of this platform, we extended our studies to a pilot cohort of two nonhuman primates (NHPs) that underwent fasciocutaneous radial forearm flap (RFF) transplantation, a clinically relevant vascularized composite allotransplantation (VCA) model that enables longitudinal monitoring of rejection. These studies represent a proof-of-concept progression from the rodent experiments and utilized both a clinical-grade PCE-NE formulation and the clinically approved FLUOBEAM imaging system. A single intravenous dose of PCE-NE (1 mL/kg) was administered on postoperative day (POD) 0 to evaluate the persistence and sensitivity of the imaging signal under a minimal dosing regimen. Despite administration of only a single dose, robust NIRF signal was detectable throughout the monitoring period and persisted for 7-8 days post-transplantation. Both animals subsequently developed acute rejection requiring flap explantation on POD8 and POD7 (NHP A and NHP B, respectively). Importantly, NIRF signal quantification was performed using the same spatially resolved region-of-interest (ROI) methodology established in the rodent studies, enabling direct cross-species comparison of inflammatory signal dynamics. Collectively, these findings demonstrate successful translation of the imaging platform from small-animal models to a clinically relevant large-animal VCA model and support future investigations evaluating extended dosing regimens, longitudinal monitoring capabilities, and applications in chronic rejection. The successful translation of this approach to a clinically relevant NHP model highlights one of its major strengths: both imaging modalities possess clear and feasible pathways toward clinical implementation in VCA surveillance. Importantly, this platform is complementary to standard immunosuppressive regimens, which were not utilized as a treatment condition in this pilot cohort, and the clinical-grade nanoimaging agent does not induce macrophage activation or exacerbate inflammatory responses, thereby preserving native immune behavior during longitudinal imaging studies. Future studies will investigate the utility of this platform in chronic rejection models and under clinically relevant immunosuppressive treatment paradigms to define how macrophage trafficking and imaging signatures evolve across distinct therapeutic conditions. Such studies will enable direct comparisons between treatment cohorts and provide mechanistic insight into the interplay between immunosuppression, macrophage-mediated inflammation, and graft rejection dynamics over extended post-transplant periods.

A strength of the presented approach is that these imaging modalities have a clinically translatable path in VCA. NIRF imaging is already used in clinical surgical environments for perfusion and tissue assessment; importantly, portable clinical NIRF systems (e.g., SPY and FLUOBEAM) are compatible with point-of-care workflows and could be adapted for rapid superficial graft surveillance without requiring advanced radiology infrastructure. However, current clinical NIRF imaging methods lack quantitative algorithms applicable for monitoring inflammation in patients. The spatially resolved NIRF signal quantification method presented here addresses this gap by enabling accurate assessment of rejection and supporting longitudinal monitoring of therapeutic response to both local and systemic immunosuppression in VCA. In a translational VCA workflow, our PCE-NE NIRF imaging technology could therefore serve not only as a standalone bedside monitoring tool but also as a sensitive triage trigger for targeted ^19^F MRI to localize deeper tissue inflammation and guide biopsy toward signal-dense compartments that are not reliably sampled by protocol skin biopsies^2^, ^23^. MRI scanners can be configured for 19F MRI with the appropriate hardware and radio-frequency coil, thus 19F MRI can be readily added on to existing clinical imaging protocols.

Finally, the clinical motivation extends beyond diagnosis alone. Breakthrough rejection related to medication nonadherence remains a significant cause of AR and graft loss in transplant recipients^41^. A practical, noninvasive surveillance method that can detect early immune activation, before obvious skin breakdown, could support earlier intervention and may provide an objective adjunct when adherence is uncertain. While this study did not directly test treatment response or adherence monitoring, it establishes a platform that is conceptually aligned with these clinical use cases.

The path to clinical translation of nanomedicines, including diagnostic probes, requires robust, scalable manufacturing and quality control. By utilizing a quality-by-design (QbD) framework and multilinear regression modeling we successfully scaled up production of PFC nanoemulsions to 1 L with strictly defined critical quality attributes, narrow droplet size, robust colloidal stability, and stable fluorescence signal under physiologic conditions^42^-^44^.

Taken together, the combined considerations of (i) established feasibility of PFC labeling and ^19^F cell tracking, (ii) durable label persistence enabling longitudinal surveillance, and (iii) scalable, QbD-compatible manufacturing pathways support clinical plausibility of the approach while still requiring appropriate regulatory development steps. This macrophage-targeted imaging technology could be adapted for longitudinal, noninvasive monitoring of whole-eye transplant rejection. Following systemic administration, the PCE-NE would label macrophages recruited during early alloimmune activation, enabling rapid NIRF surveillance of accessible ocular and periocular tissues, while ^19^F MRI could provide depth-resolved assessment of inflammation within deeper graft compartments. By limiting prolonged inflammatory injury to the retina, optic nerve, vasculature, and other critical ocular structures, this approach could improve graft survival and increase the likelihood of functional recovery after whole-eye transplantation.

Several limitations warrant consideration. First, this study establishes a primary rodent VCA model and introduces a pilot NHP cohort, and immune kinetics and tissue scale differ from human transplantation^23^. Second, although CD68 colocalization supports macrophage-associated uptake, off-target uptake by other phagocytic populations cannot be excluded^19^, ^20^. Third, early postoperative signals may reflect mixed injury/IRI inflammation and neutrophil-macrophage cooperation^19^, necessitating careful interpretation of POD0-POD2 imaging as baseline inflammatory context rather than rejection specificity. Fourth, NIRF remains limited by depth penetration; while ^19^F MRI addresses depth, clinical sensitivity thresholds and acquisition time tradeoffs will require optimization for human workflows. Finally, this work is focused on AR—consistent with available Banff criteria—but does not establish detection of CR, for which grading remains limited and an active area of consensus development^2^, ^19^, ^24^, ^25^. Additionally, the present study did not evaluate systemic clearance kinetics, blood chemistry, quantitative organ biodistribution following repeated PCE-NE administration, or the effects of repeated probe exposure on macrophage function. These parameters will require evaluation in future studies of repeated-dose imaging.

Priorities for future work include: (i) defining quantitative relationships between NIRF and ^19^F signal and absolute macrophage burden across compartments, (ii) assessing nanoimaging probe uptake by macrophage subsets and activation states, (iii) evaluating performance of nanoimaging probe in recurrent AR, CR and immunosuppressive treatment-response paradigms^2^, ^38^, ^45^, ^46^, and (iv) implementing clinically feasible ^19^F acquisition workflows embedded within standard MRI protocols. Given the emerging interest in macrophage-associated rejection biomarkers in VCA^36^, combining sequential NIRF imaging with serum biomarker panels represents another promising translational direction.

In summary, we demonstrate that a macrophage-targeted multimodal PCE-NE platform enables live longitudinal NIRF imaging and depth-resolved ^19^F MRI mapping *ex vivo* of AR across both superficial and deep graft compartments, with spatial alignment to CD68⁺ macrophage infiltration and Banff-graded histopathology. Although macrophage-associated PCE-NE uptake provides a biologically relevant imaging readout of rejection-associated inflammation, macrophage recruitment is not exclusive to allograft rejection and may also occur in response to ischemia-reperfusion injury, surgical inflammation, or other inflammatory processes. Imaging findings should therefore be interpreted within the transplant context together with clinical and histopathological findings. Single-dose administration establishes baseline kinetics, while repeated dosing captures sequential macrophage recruitment and improves tissue-scale resolution. By combining clinically applicable NIRF imaging with quantitative MRI depth mapping, this approach creates a noninvasive immune surveillance framework in VCA and a translational platform adaptable to broader alloimmune injury phenotypes.

## Supplementary Material

Supplementary methods, figures and table.

## Figures and Tables

**Figure 1:**
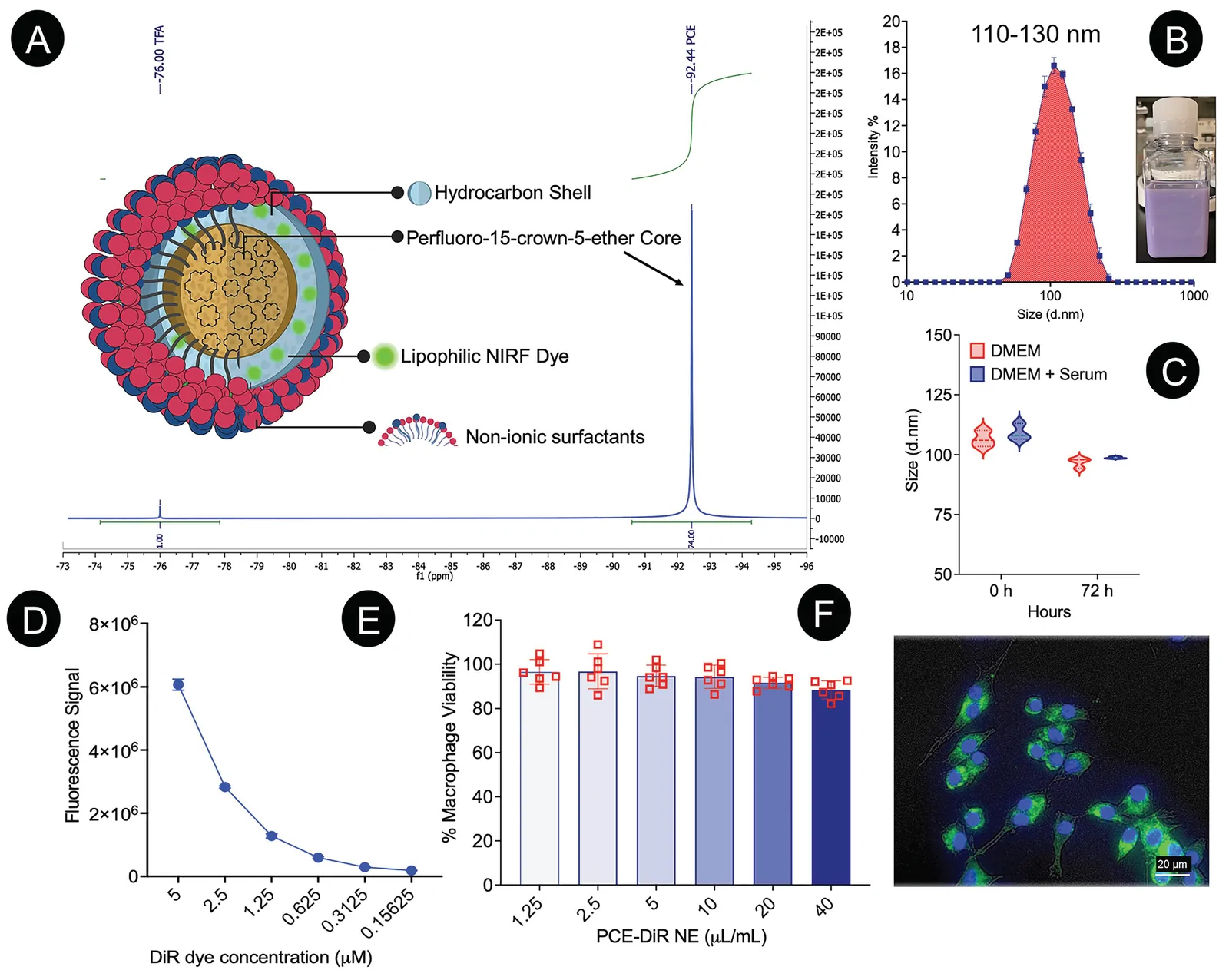
** Multimodal nanoimaging probe PCE-NE *in vitro* characterization. (A)** Schematic illustration of the triphasic PCE-NE particle composed of a perfluoro-15-crown-5 ether (PCE) core, triglyceride (hydrocarbon) shell incorporating a lipophilic NIRF dye, and a non-ionic surfactant surface; representative ^19^F NMR spectrum for PCE with a signature 20-equivalent fluorine peak at -91.5 ppm. **(B)** Overlay of the intensity-weighted droplet size distribution measured on Day 1, demonstrating a narrow diameter range of 110-130 nm. **(C)** Size measurements by DLS following 72 h incubation at 37 °C in culture medium supplemented with 20% bovine serum. **(D)** NIRF signal measurements across decreasing concentration of PCE-NE on LI-COR Odyssey. **(E)** Cell viability assessed by CellTiter-Glo Luminescence-based assay of RAW 264.7 macrophages exposed to increasing concentrations of PCE-NE for 24 h (mean ± SD, n = 6), demonstrating no measurable viability loss across the tested dose range. **(F)** Representative fluorescence microscopy of RAW 264.7 macrophages following exposure to 40 µL DiR-labeled PCE-NE (n = 6), illustrating cell uptake *in vitro* (scale bar, 20 µm); PCE-NE (green) surrounds the DAPI (blue) labeled nuclei in individual cells.

**Figure 2:**
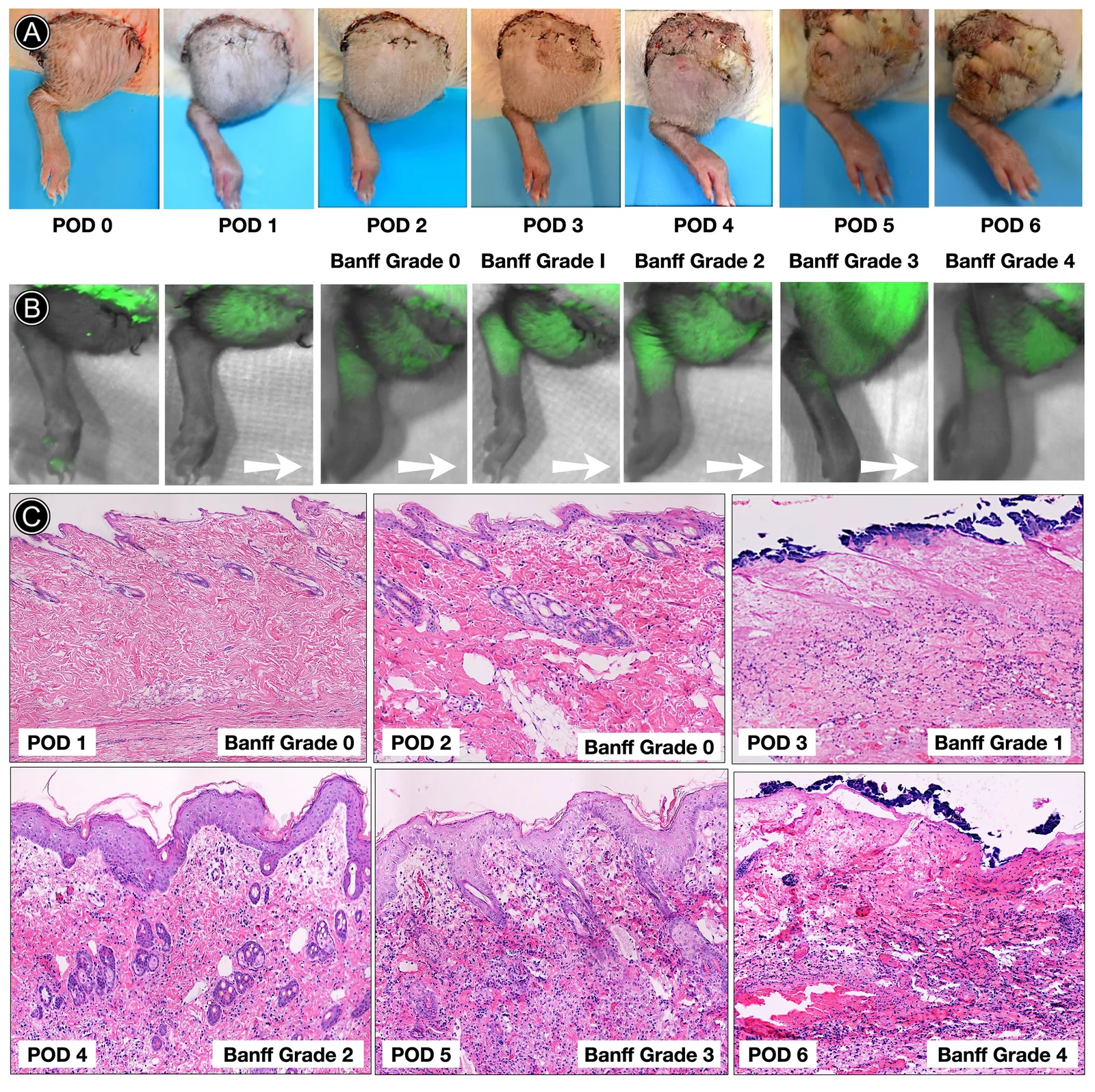
** Longitudinal single-dose PCE-NE imaging captures progressive rejection but may incompletely represent secondary macrophage waves. (A)** Serial clinical photographs of the transplanted hindlimb from POD0 through POD6 demonstrating progressive macroscopic features of acute rejection, including erythema, edema, desquamation, and tissue necrosis corresponding to escalating Banff grades. **(B)** Representative *in vivo* NIRF images acquired following a single intravenous administration of PCE-NE at POD0. Fluorescence signal progressively increases within the transplanted limb over time, with detectable focal signal as early as POD2 and continued spatial expansion through POD6. Arrows indicate sequential progression towards increasing grade of rejection.** (C)** Representative hematoxylin and eosin (H&E) sections illustrating histopathologic progression according to Banff criteria for vascularized composite allotransplantation, advancing from Grade 0 (POD1-2) to Grade I (POD3), Grade II (POD4), and Grade III-IV rejection by POD5-6.

**Figure 3:**
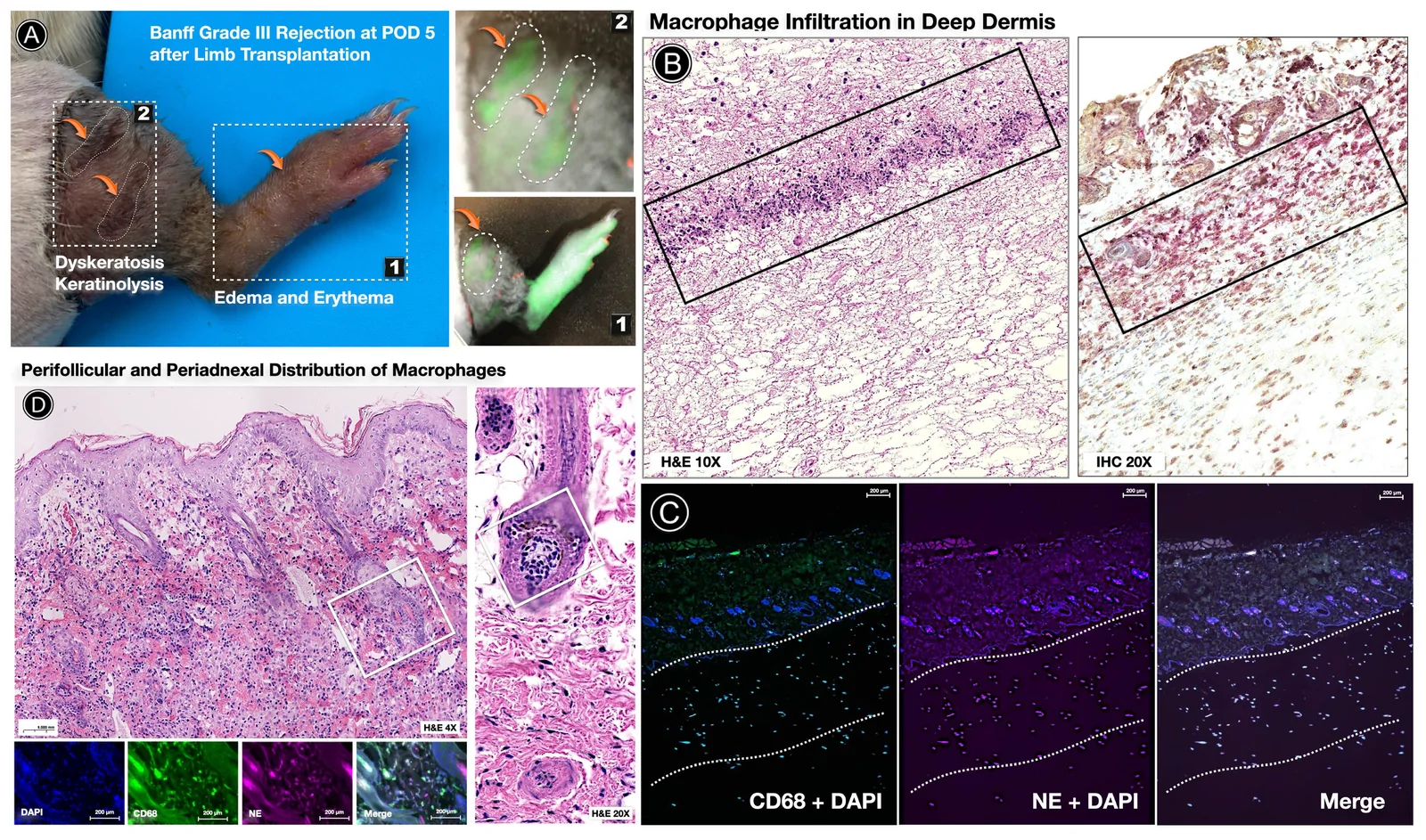
** PCE-NE fluorescence spatially corresponds with deep dermal, interface, and periadnexal CD68⁺ macrophage infiltration in Banff Grade III rejection. (A)** Gross clinical appearance of the transplanted hindlimb at POD5 demonstrating Banff Grade III rejection characterized by edema, erythema, dyskeratosis, and keratinolysis. *In vivo* NIRF imaging reveals focal and confluent fluorescence signal corresponding to clinically inflamed graft regions. **(B)** Histopathologic correlation in the same graft. Hematoxylin and eosin (H&E) staining demonstrates dense mononuclear inflammatory infiltration within the deep dermis forming interface-oriented inflammatory bands extending toward the dermal-subcutaneous junction. CD68 immunohistochemistry (red chromogen) confirms prominent macrophage accumulation within these inflammatory zones and along perivascular compartments. **(C)** Immunofluorescence analysis showing CD68 (green), PCE-NE fluorescence (magenta), and DAPI nuclear counterstain (blue). Merged images demonstrate spatial colocalization of PCE-NE signal with CD68⁺ macrophages within deep dermal interface aggregates. Regions lacking CD68⁺ cells show minimal PCE-NE fluorescence, supporting macrophage-dependent nanoemulsion uptake rather than nonspecific tissue diffusion. **(D)** Representative sections highlighting periadnexal and perifollicular inflammatory architecture characteristic of advanced rejection. H&E staining demonstrates dense inflammatory cuffs surrounding hair follicles and adnexal structures^18^. Higher magnification confirms macrophage-rich infiltrates encircling follicular units. Corresponding immunofluorescence demonstrates colocalization of PCE-NE signal with CD68⁺ macrophages within perifollicular inflammatory aggregates, indicating active nanoemulsion uptake in adnexal-associated macrophage populations.

**Figure 4:**
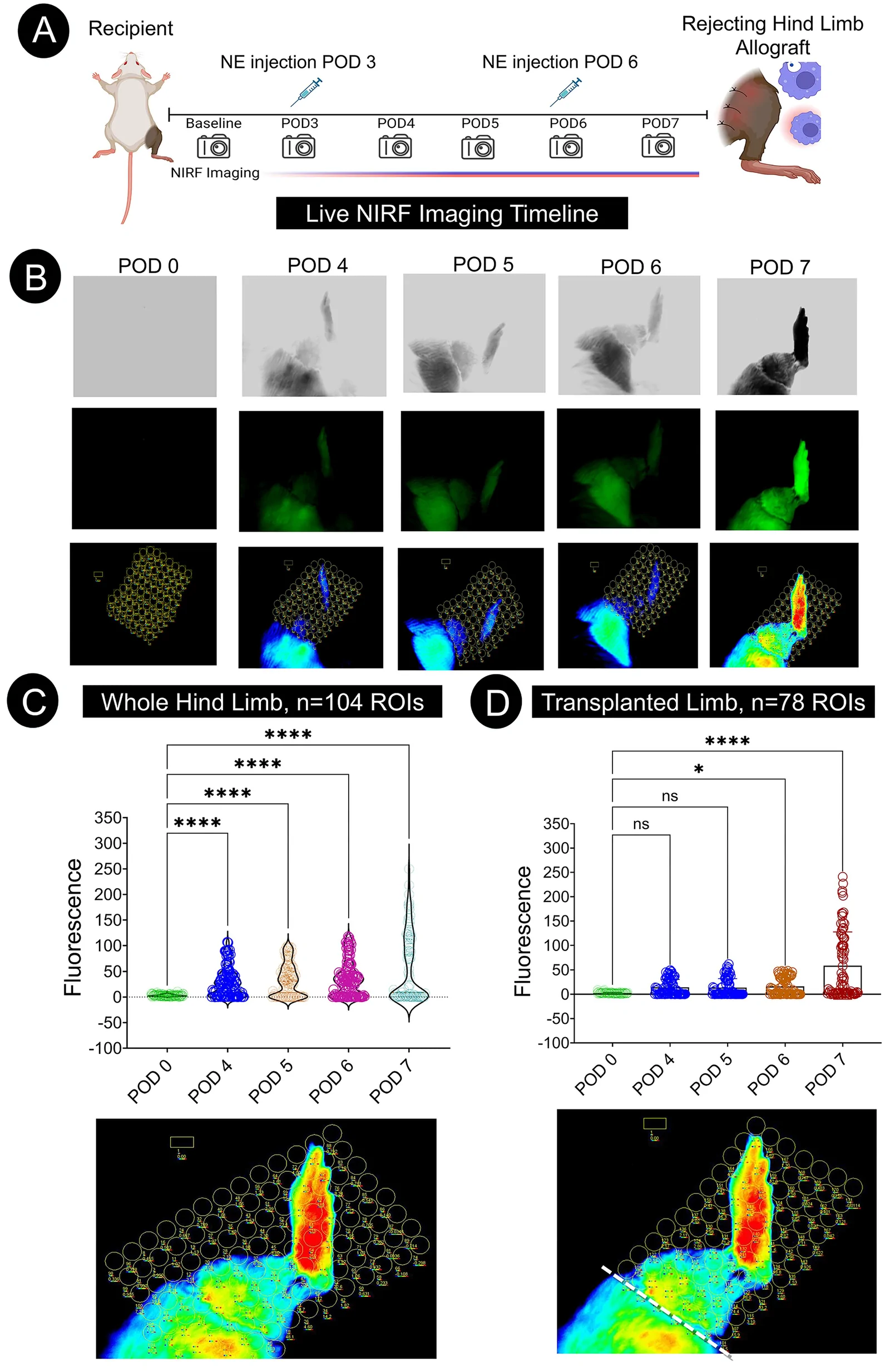
** Two-dose PCE-NE administration enables staged imaging of *in vivo* macrophage infiltration during acute rejection. (A)** Experimental timeline illustrating allogeneic hindlimb vascularized composite allotransplantation (VCA) followed by two intravenous administrations of PCE-NE at postoperative day (POD) 3 and POD6, with serial live near-infrared fluorescence (NIRF) imaging performed from post-surgical baseline through POD7. **(B)** Representative longitudinal *in vivo* NIRF images acquired using the LI-COR Pearl^®^ imager at the indicated time points from a single transplant recipient. Images are displayed as grayscale (anatomical reference), single-channel fluorescence (800 nm), and corresponding heatmaps to visualize the spatial distribution and intensity of the PCE-NE signal across the imaged hindlimb field. Progressive signal emergence is observed beginning at POD4 with increasing intensity and spatial expansion through POD7. **(C)** Quantitative analysis using a multi-sampled region-of-interest (ROI) strategy applied across the combined "donor graft-recipient stump zone", encompassing the full imaged hindlimb field (n = 104 ROIs). Fluorescence intensity increased significantly over time (one-way ANOVA; ****p < 0.0001), reflecting overall macrophage-associated signal burden across overlapping donor and proximal recipient stump compartments. Heatmap overlays illustrate heterogeneous signal distribution across sampled regions. **(D)** Signal quantification was restricted to the anatomically defined distal donor graft region distal to the suture line (n = 78 ROIs). At early time points (POD4-POD5), the signal is modestly increased over the baseline (POD0), followed by a significant increase following the second dose at POD6-POD7 (one-way ANOVA; ****p < 0.0001; *p < 0.05; ns, not significant). Heatmap representation highlights a distinct pattern of macrophage infiltration signal in the rejecting graft at endpoint (POD7).

**Figure 5:**
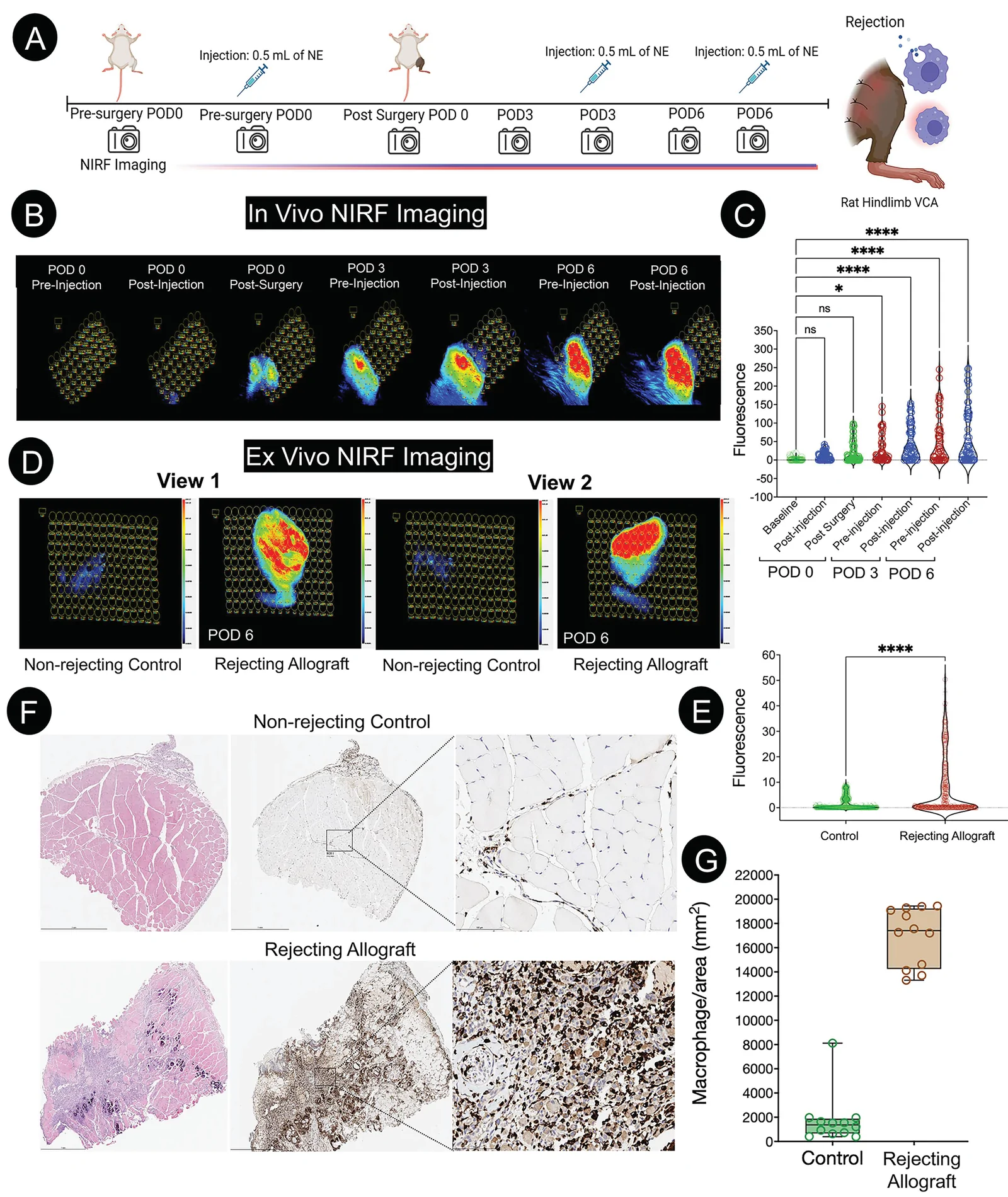
** A three-dose PCE-NE regimen enhances graft-specific *in vivo* detection of macrophage recruitment during acute rejection, with *ex vivo* and histologic validation. (A)** Schematic illustration of the experimental timeline in a rat hindlimb VCA model incorporating three intravenous PCE-NE administrations at POD0, POD3, and POD6 with serial live NIRF imaging at defined time points. **(B)** Representative longitudinal *in vivo* NIRF images acquired from a single transplant recipient using the LI-COR Pearl^®^ imager, displayed with multi-sampled ROIs to illustrate spatial distribution and temporal amplification of fluorescence signal across the allogeneic hindlimb. Minimal baseline signal is observed pre-injection. Stepwise signal escalation is evident following surgery and subsequent administrations, with maximal fluorescence intensity observed after the POD6 dose, consistent with cumulative labeling of infiltrating macrophage populations during active rejection. **(C)** Quantitative analysis of *in vivo* fluorescence using a multi-sampled ROI strategy across the imaged hindlimb field (n = 59 ROIs). Signal intensity increased progressively over time, with significant amplification observed at POD6 both pre- and post-injection (one-way ANOVA; ****p < 0.0001), reflecting accumulation of labeled macrophages during the rejection phase distinct from early post-surgical inflammation. **(D)** *Ex vivo* NIRF imaging of harvested grafts is shown in two orientations (View 1: inner surface; View 2: outer surface) to better capture surface signal distribution of a three-dimensional graft. Rejecting allografts showed strong, spatially heterogeneous fluorescence compared to a minimal background signal in non-rejecting controls. Multi-sampled ROI overlays illustrate intragraft heterogeneity in signal intensity. **(E)** Quantitative multi-sampled ROI analysis of *ex vivo* fluorescence intensity (n = 300 ROIs) demonstrating significantly greater signal in rejecting allografts compared to non-rejecting controls (one-way ANOVA; ****p < 0.0001), confirming robust graft-localized PCE-NE-labeled macrophage accumulation. **(F)** Representative histologic sections and CD68 immunostaining from non-rejecting and rejecting allografts. The rejecting grafts exhibit dense CD68⁺ macrophage infiltration compared to sparse staining in controls. **(G)** Quantitative analysis of CD68⁺ macrophage area density (cells/mm²) demonstrating significant elevation in rejecting allografts relative to non-rejecting controls (****p < 0.0001).

**Figure 6:**
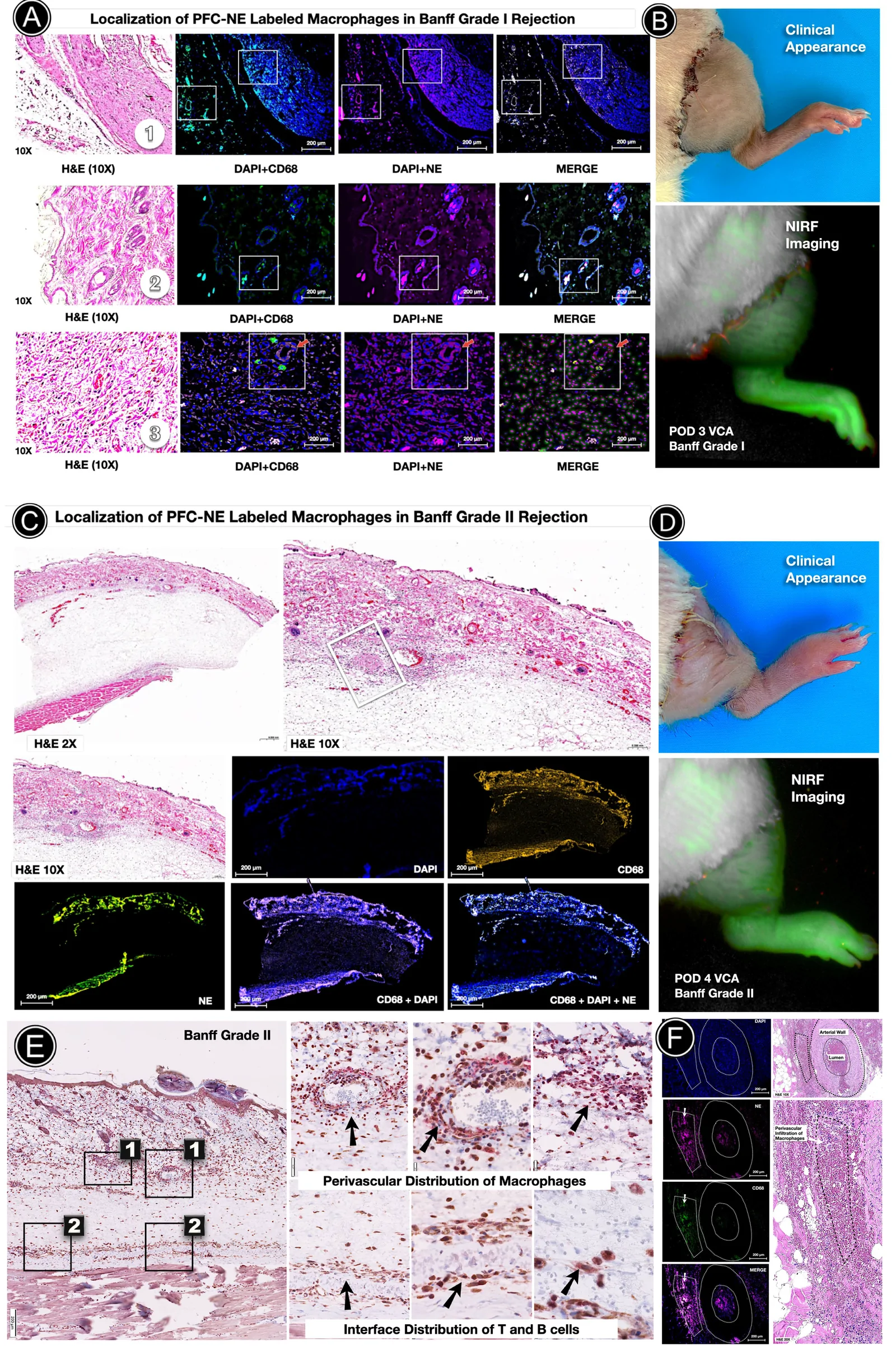
** Early Banff Grade I-II macrophage detection by PCE-NE NIRF imaging. (A)** Localization of PCE-NE-labeled macrophages in Banff Grade I rejection (POD3). Deep biopsy sections from muscle (1) and deep dermis (3) demonstrate focal macrophage infiltration surrounding capillaries and extending toward superficial epidermal and adnexal compartments (2). Immunofluorescence shows CD68 (green) and PCE-NE signal (magenta) with DAPI counterstain (blue), demonstrating spatial colocalization across deep dermal and epidermal-adnexal regions. **(B)** Corresponding clinical and *in vivo* NIRF images at Banff Grade I rejection. Despite subtle clinical findings limited to mild erythema, NIRF signal within the skin compartment is clearly detectable, indicating early macrophage-associated fluorescence preceding overt tissue injury. **(C)** Full-thickness limb sections in Banff Grade II rejection demonstrating expanded inflammatory architecture. H&E reveals deep dermal inflammatory infiltration with involvement of a dermal arteriole. **(D)** Clinical appearance and corresponding *in vivo* NIRF imaging at Banff Grade II rejection. Fluorescence remains robust within the graft despite modest external clinical changes, highlighting the sensitivity of PCE-NE-based detection. **(E)** CD68 immunohistochemistry (red chromogen) confirms macrophage accumulation within perivascular zones and deeper dermal compartments in Banff Grade II rejection. **(F)** High-resolution immunofluorescence demonstrating unequivocal colocalization of PCE-NE signal with CD68⁺ macrophages in perivascular regions surrounding a dermal arteriole, supporting macrophage-associated nanoemulsion uptake in early-to-intermediate rejection stages.

**Figure 7:**
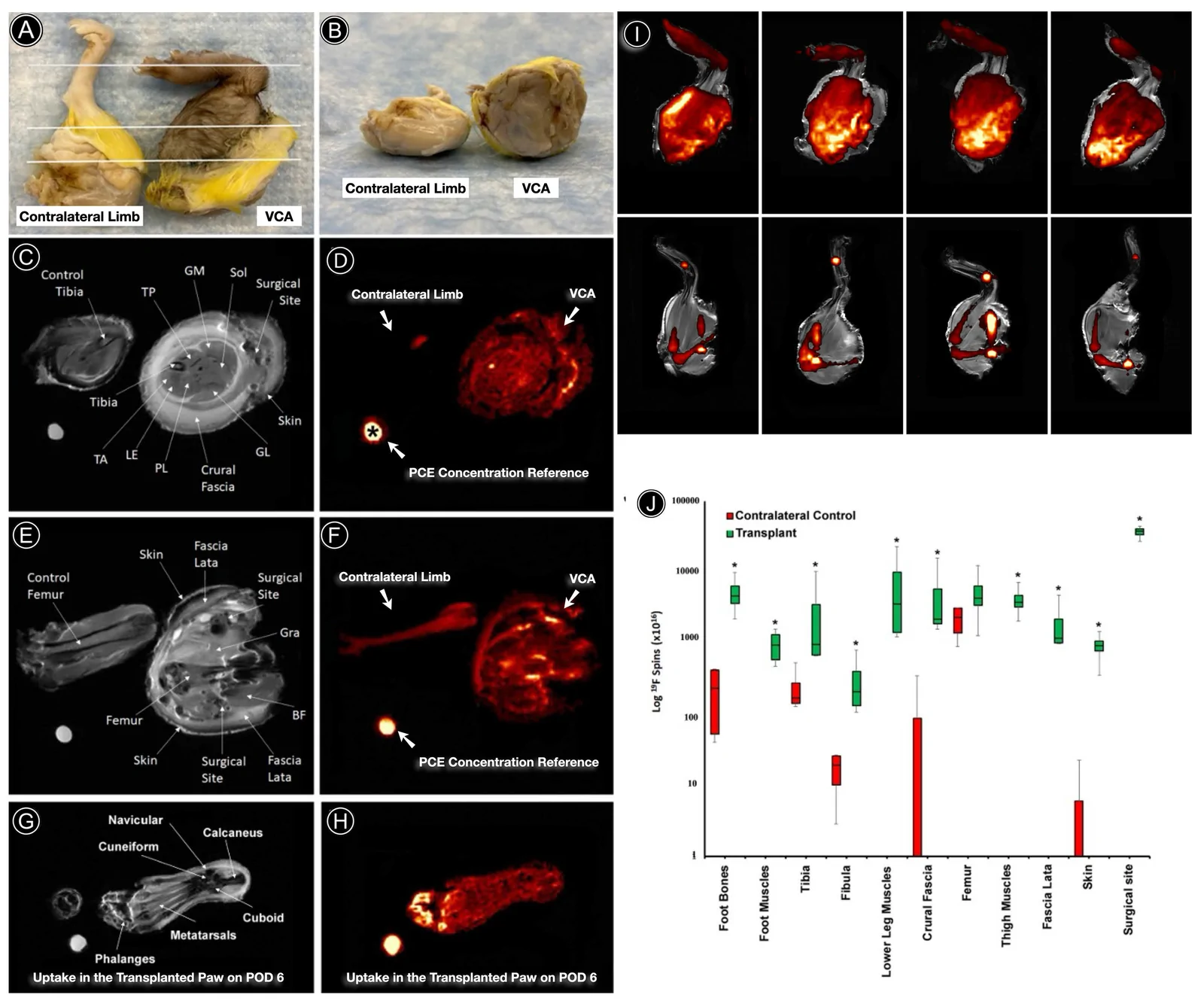
** Whole-limb ^19^F MRI demonstrates graft-wide PCE-NE accumulation across multiple tissue compartments during rejection. (A, B)** Photographs of contralateral control and VCA allograft limbs illustrating orientation within the MRI scanner (n = 4). White lines indicate approximate locations of proximal, mid, and distal image slices shown in subsequent panels. **(C = mid, G = distal, E = proximal)** Representative anatomical (¹H) MR images from proximal, mid, and distal axial slices of control and transplant limbs with major anatomical structures labeled, including tibia, fibula, tibialis anterior (TA), tibialis posterior (TP), gastrocnemius medial (GM), gastrocnemius lateral (GL), soleus (Sol), long extensor (LE), peroneus longus (PL), gracilis (Gra), biceps femoris (BF), fascia compartments, and surgical site. **(D, F, H)** Corresponding ^19^F MR images rendered in hot-iron pseudocolor showing spatial distribution of PCE-NE signal within the same slices. In transplanted limbs, fluorine signal is widely distributed throughout muscle compartments, fascia, skin, foot musculature, bone marrow, and the surgical interface. In contralateral control limbs, ^19^F signal is minimal and largely confined to physiologic bone marrow compartments of the tibia, fibula, and femur. The PCE concentration reference standard used for quantification is indicated by “*”. **(I)** Pseudo-sagittal images of ^19^F signal distribution from four transplant-contralateral limb pairs. Transplanted limbs (top row) demonstrate extensive graft-wide fluorine accumulation compared with limited marrow-restricted signal in contralateral controls (bottom row). Anatomical background (grayscale) represents a mid 2-mm ¹H slice, while ^19^F signal reflects full-slice acquisition of the entire limb volume. **(J)** Box-and-whisker plot (log scale) of compartment-specific fluorine quantification (N=4 transplant, N=4 contralateral). Significantly greater ^19^F signal was detected in transplant limbs across multiple compartments, including foot bones, foot muscles, tibia, fibula, lower leg muscles, crural fascia, thigh muscles, fascia lata, skin, and surgical site (*p < 0.05). The surgical site demonstrated the highest fluorine signal intensity among compartments. Box represents interquartile ranges, while the line is the median, top whisker is the maximum and the bottom whisker is the minimum.

**Figure 8:**
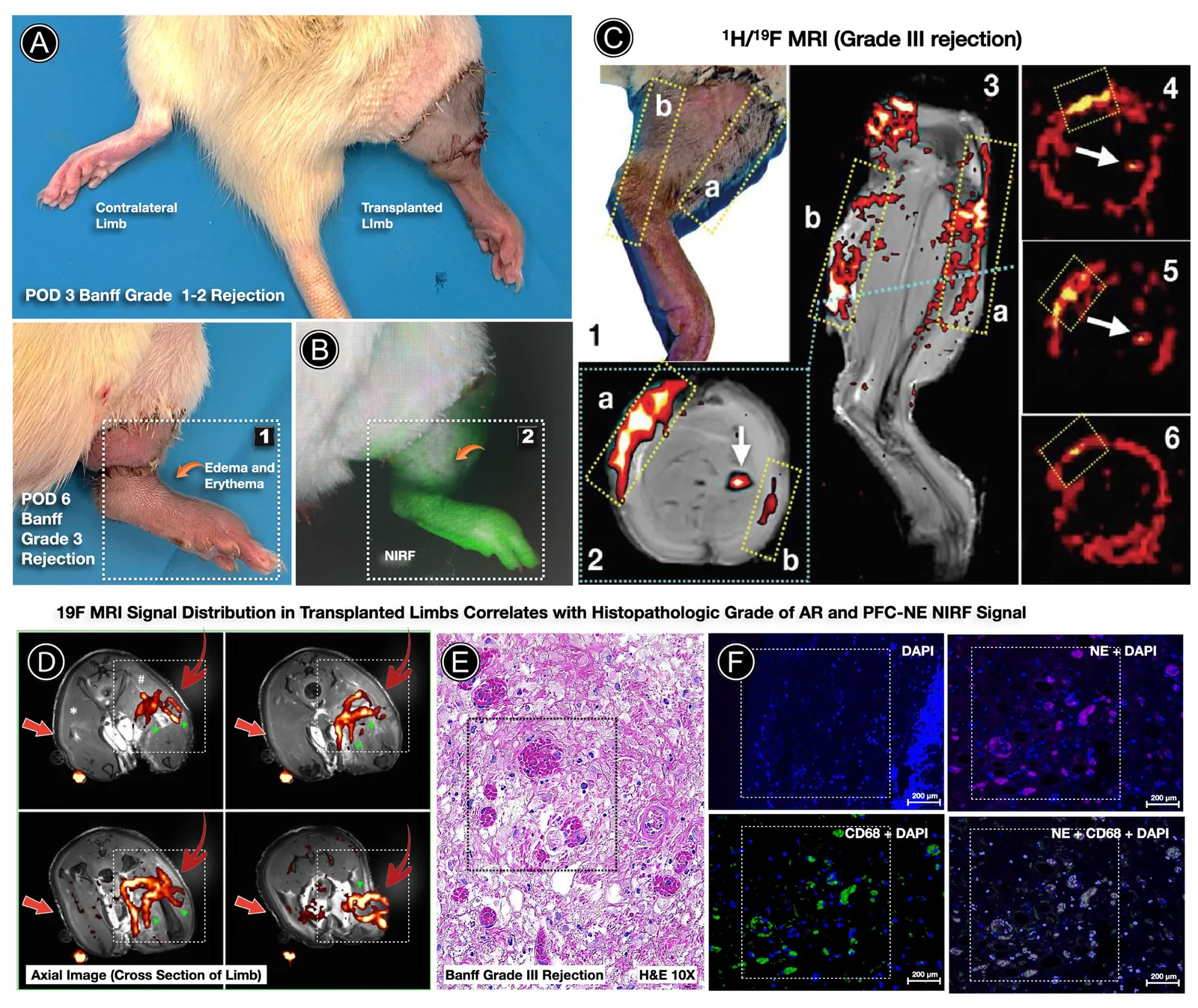
** Multimodal imaging with PCE-NE integrates clinical, NIRF, and ^19^F MRI mapping of macrophage infiltration during acute rejection. (A)** Clinical appearance of the transplanted hindlimb during evolving acute rejection. At POD3 (Banff Grade I-II), subtle erythema and edema are present despite preserved gross limb architecture. By POD6 (Banff Grade III), more pronounced inflammatory changes are evident. The contralateral limb serves as internal control. **(B)** *In vivo* NIRF imaging following PCE-NE administration demonstrates robust fluorescence signal within the transplanted limb despite subtle clinical findings at earlier Banff grades. Signal localizes predominantly to the skin and subcutaneous compartments and intensifies with advancing rejection severity. **(C)** Combined ¹H/^19^F MRI of rejecting limbs (Banff Grade III) demonstrates depth-resolved PCE-NE signal distribution. Coronal and axial slice-selective imaging reveal fluorine signal (rendered in hot-iron pseudo-color) extending across superficial dermis, fascia, and deep muscular compartments. Dashed regions correspond to areas of clinical inflammation and NIRF signal localization. Axial reconstructions (panels 4-6) demonstrate pericompartmental and perivascular signal patterns within deep tissue planes. **(D)** Representative axial ¹H/^19^F MRI sections of transplant versus contralateral limbs demonstrate marked compartmental fluorine signal enrichment within the rejecting graft, including fascia, muscle groups, and peri-surgical regions, with minimal signal confined to marrow compartments in contralateral controls. **(E)** Gold-standard histopathology (H&E, 10×) confirms Banff Grade III rejection characterized by dense mononuclear infiltration within dermal and deep soft tissue compartments corresponding spatially to MRI signal-positive regions. **(F)** Immunofluorescence validation demonstrates colocalization of PCE-NE signal with CD68⁺ macrophages within inflamed tissue compartments. DAPI (blue), CD68 (green), PCE-NE (magenta), and merged images confirm cellular uptake-dependent signal localization rather than diffuse extracellular deposition.

**Figure 9:**
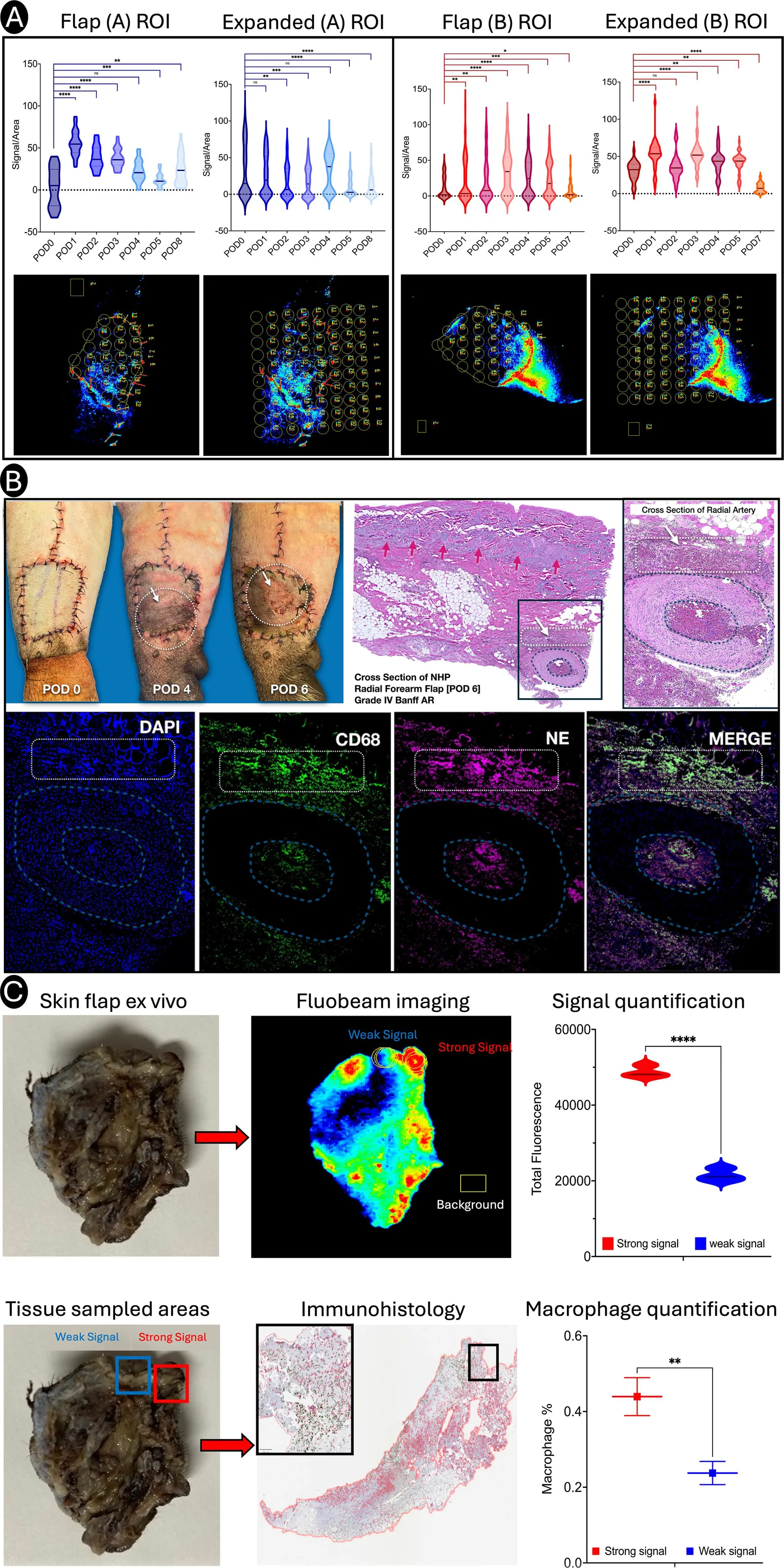
** Longitudinal *in vivo* and *ex vivo* fluorescence imaging of acute rejection in nonhuman primate radial forearm flap transplantation and correlation with macrophage infiltration. (A)** Quantification of longitudinal *in vivo* fluorescence signal in both nonhuman primates (NHPs) following radial forearm flap transplantation. Fluorescence intensity was measured on postoperative days (POD) 0, 1, 2, 3, 4, 5, and 7 (NHP B only) and POD8 (NHP A only). The first and third columns show quantification using a flap-only ROI (NHP A and NHP B, respectively), and the second and fourth columns show quantification using an expanded ROI encompassing the flap and surrounding perisurgical tissue; representative ROI placements are shown below each plot. Statistical significance was determined using an unpaired two-tailed t-test comparing fluorescence intensity at POD0 with each subsequent postoperative time point. **(B)** Histopathological and immunofluorescence characterization of acute rejection in NHP A at POD8. The upper left panel shows clinical presentation of Grade III-IV acute rejection. The upper right panel displays hematoxylin and eosin (H&E) staining (2×, left; 20×, right), demonstrating dense inflammatory infiltrates within the deep dermis (red arrows) and perivascular regions (white hashed area and white arrow). The radial artery is outlined by black hashed lines. The lower panels show immunofluorescence staining demonstrating colocalization of nanoemulsion (NE) signal with CD68-positive macrophages within perivascular inflammatory infiltrates, confirming macrophage accumulation in regions of deep acute rejection and perivasculitis. CD68- and NE-positive cells are also observed within the arterial lumen. **(C)** *Ex vivo* fluorescence imaging and macrophage quantification of rejected flap tissue from NHP A at POD8. The upper left panel shows the excised rejected flap. The upper middle panel presents FLUOBEAM imaging of the explanted tissue, with triplicate circular ROIs placed within regions of weak and strong fluorescence signal. The upper right panel shows quantification of fluorescence intensity within these ROIs; statistical significance was assessed using an unpaired two-tailed t-test. The lower left panel identifies tissue sampling locations from weak-signal (blue box) and strong-signal (red box) regions. The lower middle panel shows macrophage detection and classification performed using QuPath software, with green cells classified as macrophages and red cells classified as non-macrophage cells. The lower right panel presents quantitative comparison of macrophage percentage between weak- and strong-signal regions, with statistical significance determined using an unpaired two-tailed t-test.

## Data Availability

The numerical data underlying this study are available from the corresponding authors upon reasonable request and in accordance with institutional policies and procedures.

## Abbreviations

CD68: Cluster of differentiation 68
MMP3: Matrix metalloproteinase-3
MRI: Magnetic resonance imaging
NE: Nanoemulsion
NIRF: Near-infrared fluorescence
PCE: Perfluoro-15-crown-5 ether
PFC: Perfluorocarbon
QbD: Quality-by-Design
ROI: Region of interest
VCA: Vascularized composite allotransplantation

## References

[1] Cendales LC, Kanitakis J, Schneeberger S, Burns C, Ruiz P, Landin L (2008). The Banff 2007 working classification of skin-containing composite tissue allograft pathology. Am J Transplant.

[2] Gorantla VS, Demetris AJ (2011). Acute and chronic rejection in upper extremity transplantation: what have we learned?. Hand Clin.

[3] Hautz T, Zelger BG, Weissenbacher A, Zelger B, Brandacher G, Landin L (2013). Standardizing skin biopsy sampling to assess rejection in vascularized composite allotransplantation. Clin Transplant.

[4] Sarhane KA, Khalifian S, Ibrahim Z, Cooney DS, Hautz T, Lee WP (2014). Diagnosing skin rejection in vascularized composite allotransplantation: advances and challenges. Clin Transplant.

[5] Jiang X, Tian W, Sung YK, Qian J, Nicolls MR (2014). Macrophages in solid organ transplantation. Vasc Cell.

[6] Salehi S, Reed EF (2015). The divergent roles of macrophages in solid organ transplantation. Curr Opin Organ Transplant.

[7] Vichare R, Kulahci Y, McCallin R, Zor F, Selek FN, Liu L (2025). Theranostic nanoemulsions suppress macrophage-mediated acute inflammation in rats. J Nanobiotechnology.

[8] Nichols JM, Crelli CV, Liu L, Pham HV, Janjic JM, Shepherd AJ (2021). Tracking macrophages in diabetic neuropathy with two-color nanoemulsions for near-infrared fluorescent imaging and microscopy. J Neuroinflammation.

[9] Vasudeva K, Andersen K, Zeyzus-Johns B, Hitchens TK, Patel SK, Balducci A (2014). Imaging neuroinflammation in vivo in a neuropathic pain rat model with near-infrared fluorescence and (1)(9)F magnetic resonance. PLoS One.

[10] Hitchens TK, Ye Q, Eytan DF, Janjic JM, Ahrens ET, Ho C (2011). 19F MRI detection of acute allograft rejection with in vivo perfluorocarbon labeling of immune cells. Magn Reson Med.

[11] Herneisey M, Janjic JM (2023). Multiple Linear Regression Predictive Modeling of Colloidal and Fluorescence Stability of Theranostic Perfluorocarbon Nanoemulsions. Pharmaceutics.

[12] Cortez I, Gaffney CM, Vichare R, Crelli CV, Liu L, Lee E (2024). Neuromuscular Polytrauma Pain is Resolved by Macrophage COX-2 Nanoimmunomodulation. Int J Nanomedicine.

[13] Cortez I, Gaffney CM, Crelli CV, Lee E, Nichols JM, Pham HV (2024). Sustained pain and macrophage infiltration in a mouse muscle contusion model. Muscle Nerve.

[14] Liu L, Karagoz H, Herneisey M, Zor F, Komatsu T, Loftus S (2020). Sex Differences Revealed in a Mouse CFA Inflammation Model with Macrophage Targeted Nanotheranostics. Theranostics.

[15] Saleem M, Deal B, Nehl E, Janjic JM, Pollock JA (2019). Nanomedicine-driven neuropathic pain relief in a rat model is associated with macrophage polarity and mast cell activation. Acta Neuropathol Commun.

[16] Saleem M, Stevens AM, Deal B, Liu L, Janjic J, Pollock JA (2019). A New Best Practice for Validating Tail Vein Injections in Rat with Near-infrared-Labeled Agents. J Vis Exp.

[17] Janjic JM, Vasudeva K, Saleem M, Stevens A, Liu L, Patel S (2018). Low-dose NSAIDs reduce pain via macrophage targeted nanoemulsion delivery to neuroinflammation of the sciatic nerve in rat. J Neuroimmunol.

[18] Sun JA, Adil A, Biniazan F, Haykal S (2024). Immunogenicity and tolerance induction in vascularized composite allotransplantation. Front Transplant.

[19] Bouchery T, Harris N (2019). Neutrophil-macrophage cooperation and its impact on tissue repair. Immunol Cell Biol.

[20] Scozzi D, Ibrahim M, Menna C, Krupnick AS, Kreisel D, Gelman AE (2017). The Role of Neutrophils in Transplanted Organs. Am J Transplant.

[21] Schneider M, Cardones AR, Selim MA, Cendales LC (2016). Vascularized composite allotransplantation: a closer look at the banff working classification. Transpl Int.

[22] Etra JW, Grzelak MJ, Fidder SAJ, Kolegraff K, Bonawitz S, Shores J (2019). A Skin Rejection Grading System for Vascularized Composite Allotransplantation in a Preclinical Large Animal Model. Transplantation.

[23] Starzl R, Brandacher G, Lee WP, Carbonell J, Zhang W, Schnider J (2013). Review of the early diagnoses and assessment of rejection in vascularized composite allotransplantation. Clin Dev Immunol.

[24] Song EY, Barrow BE, Cendales LC (2024). Vascular changes in vascularized composite allotransplantation. Curr Opin Organ Transplant.

[25] Cendales LC, Farris AB, Rosales I, Elder D, Gamboa-Dominguez A, Gelb B (2024). Banff 2022 Vascularized Composite Allotransplantation Meeting Report: Diagnostic criteria for vascular changes. Am J Transplant.

[26] Ordikhani F, Pothula V, Sanchez-Tarjuelo R, Jordan S, Ochando J (2020). Macrophages in Organ Transplantation. Front Immunol.

[27] Kollar B, Uffing A, Borges TJ, Shubin AV, Aoyama BT, Dagot C (2019). MMP3 Is a Non-invasive Biomarker of Rejection in Skin-Bearing Vascularized Composite Allotransplantation: A Multicenter Validation Study. Front Immunol.

[28] Haug V, Kollar B, Obed D, Kiwanuka H, Turk M, Wo L (2019). The Evolving Clinical Presentation of Acute Rejection in Facial Transplantation. JAMA Facial Plast Surg.

[29] van Heeswijk RB, Bauer WR, Bonner F, Janjic JM, Mulder WJM, Schreiber LM (2023). Cardiovascular Molecular Imaging With Fluorine-19 MRI: The Road to the Clinic. Circ Cardiovasc Imaging.

[30] Temme S, Kleimann P, Tiren ZB, Bouvain P, Zielinski A, Dollmeyer W (2025). Imaging of Thromboinflammation by Multispectral (19)F MRI. Int J Mol Sci.

[31] Ahrens ET, Helfer BM, O'Hanlon CF, Lister DR, Bykowski JL, Messer K (2023). Method for estimation of apoptotic cell fraction of cytotherapy using in vivo fluorine-19 magnetic resonance: pilot study in a patient with head and neck carcinoma receiving tumor-infiltrating lymphocytes labeled with perfluorocarbon nanoemulsion. J Immunother Cancer.

[32] Ahrens ET, Helfer BM, O'Hanlon CF, Schirda C (2014). Clinical cell therapy imaging using a perfluorocarbon tracer and fluorine-19 MRI. Magn Reson Med.

[33] Flogel U, Su S, Kreideweiss I, Ding Z, Galbarz L, Fu J (2011). Noninvasive detection of graft rejection by in vivo (19) F MRI in the early stage. Am J Transplant.

[34] Balducci A, Wen Y, Zhang Y, Helfer BM, Hitchens TK, Meng WS (2013). A novel probe for the non-invasive detection of tumor-associated inflammation. Oncoimmunology.

[35] Li J, Li C, Zhuang Q, Peng B, Zhu Y, Ye Q (2019). The Evolving Roles of Macrophages in Organ Transplantation. J Immunol Res.

[36] Cheng HY, Anggelia MR, Lin CH (2025). Unraveling the Roles of Macrophages in Vascularized Composite Allotransplantation. Biomedicines.

[37] Edtinger K, Yang X, Uehara H, Tullius SG (2014). Current status of vascularized composite tissue allotransplantation. Burns Trauma.

[38] Honeyman C, Stark HL, Fries CA, Gorantla VS, Davis MR, Giele H (2021). Vascularised composite allotransplantation in solid organ transplant recipients: A systematic review. J Plast Reconstr Aesthet Surg.

[39] Kueckelhaus M, Fischer S, Seyda M, Bueno EM, Aycart MA, Alhefzi M (2016). Vascularized composite allotransplantation: current standards and novel approaches to prevent acute rejection and chronic allograft deterioration. Transpl Int.

[40] Kollar B, Shubin A, Borges TJ, Tasigiorgos S, Win TS, Lian CG (2018). Increased levels of circulating MMP3 correlate with severe rejection in face transplantation. Sci Rep.

[41] Bendersky VA, Saha A, Sidoti CN, Ferzola A, Downey M, Ruck JM (2023). Factors impacting the medication "Adherence Landscape" for transplant patients. Clin Transplant.

[42] Herneisey M, Janjic JM (2023). Multiple linear regression predictive modeling of colloidal and fluorescence stability of theranostic perfluorocarbon nanoemulsions. Pharmaceutics.

[43] Janjic JM, McCallin R, Liu L, Crelli C, Das AC, Troidle A (2023). In vitro Quality Assessments of Perfluorocarbon Nanoemulsions for Near-infrared Fluorescence Imaging of Inflammation in Preclinical Models. Bio Protoc.

[44] Herneisey M, Liu L, Lambert E, Schmitz N, Loftus S, Janjic JM (2019). Development of Theranostic Perfluorocarbon Nanoemulsions as a Model Non-Opioid Pain Nanomedicine Using a Quality by Design (QbD) Approach. AAPS PharmSciTech.

[45] Schneeberger S, Gorantla VS, Hautz T, Pulikkottil B, Margreiter R, Lee WP (2009). Immunosuppression and rejection in human hand transplantation. Transplant Proc.

[46] Gorantla VS, Barker JH, Jones JW Jr, Prabhune K, Maldonado C, Granger DK (2000). Immunosuppressive agents in transplantation: mechanisms of action and current anti-rejection strategies. Microsurgery.

