# Supplementary material for: Longitudinal, Non-invasive Live Near-Infrared Fluorescence Imaging of Acute Vascularized Composite Allograft Rejection Validated by Ex Vivo 19F Magnetic Resonance Imaging and Histopathology
Source: Nanotheranostics. 2026 Oct 9;10:285–305. doi: 10.7150/ntno.139245 (PMC13686790; doi:10.7150/ntno.139245)
Supplement: Supplementary file 1 [file ntnov10p0285s1.pdf]

Supplemental Information

Longitudinal, Non-invasive Live Near-Infrared Fluorescence Imaging of Acute Vascularized Composite Allograft Rejection Validated by Ex Vivo ^{19}F Magnetic Resonance Imaging and Histopathology

Jelena M. Janjic[1]*, Yalcin Kulahci[2], Fatih Zor[2], Sanjida Ahmed Srishti[1], Riddhi Vichare[1], Lesley M. Foley[3], Zeynep Demir[2], Bedreddin Sazoğlu[2], Fatma Nurefsan Selek[2], Omer Dirican[2], Vidya Surti[1], Stefan Stratimirovic[1], Caitlin Crelli[1], Rebecca McCallin[1], Michele Herneisey[1], Huseyin Karagoz[2], T. Kevin Hitchens [3,4]* and Vijay S. Gorantla[2]*

[1] Nanomedicine Manufacturing Laboratory, Graduate School of Pharmaceutical Sciences and School of Pharmacy, Duquesne University, Pittsburgh, PA, USA
[2] Wake Forest Institute for Regenerative Medicine, Advocate Health – Atrium Wake Forest Baptist Medical Center, Winston-Salem, NC, USA
[3] Advanced Imaging Center, University of Pittsburgh, Pittsburgh, PA, USA
[4] Department of Neurobiology, University of Pittsburgh, Pittsburgh, PA, USA

*Co-Corresponding Authors

1. SUPPLEMENTAL MATERIALS:

The following materials were purchased and used as provided: The perfluoro-15-crown-5 ether (PCE) from Exfluor Corporation (Round Rock, TX, USA), Pluronic® P105 (#P4283) from US Biologicals (Salem, MA, USA) and Castor oil polyoxyethylene ether, Cremophor® EL from MilliporeSigma (Burlington, MA, USA). The NIRF dye: DiR'; DiIC18(7) (1,1'-Dioctadecyl-3,3,3',3'-Tetramethylindotricarbocyanine Iodide) was purchased from ThermoFisher Scientific (Waltham, MA 02451, USA). **Equipment:** Microfluidizer M110S and LM20 were purchased from Microfluidics Corporation (Westwood, MA, USA). The Pearl Trilogy small animal imaging system was purchased from LI-COR (Lincoln, NE, USA). ZetaSizer Nano from Malvern, UK was used for particle size measurements. Keyence Digital Microscope (Cranberry Twp, PA, USA) was used for immunofluorescence imaging.

2. SUPPLEMENTAL METHODS:

2.1. Nanoemulsion manufacturing and physicochemical characterization.

Nanoemulsions were manufactured using a high-pressure microfluidizer (M110S or LM20; Microfluidics, Westwood, MA) as previously described[1-3][1-3][1-3]. Triglyceride oil, perfluorocarbon (PFC), and DiR (in DMSO) were combined with Pluronic surfactant and processed to form the final emulsion. Particle size and polydispersity index were measured by dynamic light scattering (Zetasizer Nano ZS; Malvern Panalytical) following 1:80 dilution in water at 25 °C (173° scattering angle). Fluorescence was quantified using a LI-COR Odyssey M (Ex 750 nm/Em 780 nm). Serum stability was assessed by incubating nanoemulsion (1:5 v/v) in DMEM containing 20% FBS at 37 °C for 72 h, followed by DLS analysis. ^{19}F NMR spectra were acquired on a 400 MHz Bruker instrument using samples containing nanoemulsion, 0.4% TFA, and D2O; 16 scans were collected and analyzed with MestReNova software.

2.2. Fluorescence microscopy

RAW 264.7 cells were seeded at 20,000 cells per well in an 8-well chamber slide (Lab-Tek II) and left for attachment overnight. After 24 h, the existing media was removed, and the cells were treated with 40 µL of nanoemulsion for 6 h. Then cells were thoroughly washed with 1× PBS and mounted with mounting solution containing DAPI at ambient temperature before being imaged on the Keyence BZ-X microscope using Cy7 channel.

2.3. Rat Hindlimb VCA Model (BN→Lewis for AR)

Surgical Procedures: Naïve male Lewis rats aged 8 to 10 weeks, weighing 200 to 250 g were used as recipients. Fully MHC-mismatched Brown Norway (BN) rats were used as donors for allogeneic transplantations. Experiments were conducted in the AAALAC-accredited WFIRM VCA Laboratory on the 3rd floor of the Dean Building.

2.4. Orthotopic Hindlimb VCA (Syngeneic and Allogeneic Transplant)

Procedure (Figure S1):[4-6] In the donor animal, a circumferential skin incision was made in the proximal one-third of the right limb. The femoral artery and vein were dissected, clamped, and transected proximal to the superficial epigastric artery. The femoral nerve was dissected and transected 1 cm distal to the inguinal ligament. The biceps femoris muscle was transected to expose the sciatic nerve. The sciatic nerve was then transected proximal to its bifurcation, and the limb was amputated at the mid-femoral level.

The same procedure was performed in the recipient animal. Following amputation of the recipient's limb, the donor limb was attached using an intramedullary pin. All major muscle groups were sutured in anatomical alignment. The femoral vessels of the donor and recipient were anastomosed under an operating microscope using 10-0 sutures with a standard end-to-end microsurgical technique. The femoral and sciatic nerves were repaired using a conventional epineural technique[7]. This model provided a stringent platform to assess nanoimaging performance in deep tissues and complex inflammatory environments, including macrophage infiltration within muscle, nerve, and vascular compartments.

Anesthesia and Analgesia: All animals were carefully monitored for signs of pain and discomfort, including behavioral changes, lethargy, and tachypnea. Long-acting buprenorphine was administered immediately prior to surgery. Appropriate postoperative analgesia was provided to ensure comfort and minimize distress, pain, and injury. If severe complications or unrelieved pain were detected, animals were immediately removed from the study and euthanized.

Monitoring, Euthanasia and Tissue Harvest: Animals were monitored for 1–2 weeks or until reaching Banff Grade III acute rejection. Tissue samples were collected under isoflurane anesthesia. Endpoint euthanasia was performed by isoflurane overdose or earlier if animals became moribund or showed persistent pain despite analgesia. Following restoration of perfusion, 500 µL of nanoemulsion was injected intravenously into the external jugular vein of each recipient. Animals were euthanized at predefined postoperative time points according to group assignment. If severe pain or distress occurred prior to study completion, animals were euthanized to prevent unnecessary suffering. Euthanasia was performed by overdose inhalation anesthesia with isoflurane, in accordance with American Veterinary Medical Association (AVMA) guidelines. Component VCA tissues were harvested for histopathologic, immunohistochemical, and immunofluorescence analyses, and were correlated with longitudinal ^{19}F MRI imaging of graft-infiltrating macrophage populations.

2.5. Clinical Rejection Monitoring and Banff-Based Histologic Grading

Clinical evaluation of the skin component of the graft included assessment for erythema, edema, induration, scaling, desquamation, and necrosis. Clinical suspicion of rejection prompted histopathologic evaluation.[8, 9]

Acute rejection was graded according to the Banff 2007 Working Classification for skin-containing composite tissue allografts[10, 11]. Histologic evaluation was performed on hematoxylin and eosin–stained sections and included assessment of inflammatory infiltrate density and distribution (perivascular

versus interstitial), epidermal and adnexal involvement, spongiosis, lymphocyte exocytosis, keratinocyte apoptosis, dyskeratosis, and necrosis.[12-14]

- Grade 0 was defined as absent or rare inflammatory infiltrates.
- Grade I (mild) consisted of superficial perivascular mononuclear infiltration without epidermal involvement.
- Grade II (moderate) demonstrated moderate-to-severe perivascular inflammation with limited epidermal and/or adnexal involvement (spongiosis and/or exocytosis) in the absence of epithelial apoptosis or dyskeratosis.
- Grade III (severe) was characterized by dense dermal inflammation with epithelial apoptosis, dyskeratosis, and/or keratinolysis.
- Grade IV (necrotizing rejection) showed frank epidermal or dermal necrosis.

Animals reaching Banff Grade III rejection were euthanized as a humane endpoint to prevent progression to necrotizing rejection. Animals demonstrating severe distress, rapid clinical deterioration, or Grade IV necrosis were immediately euthanized irrespective of study time point.

At euthanasia, full-thickness graft tissues were harvested for histopathologic, immunohistochemical, and immunofluorescence analyses, and correlated with longitudinal imaging and immunologic data as specified in the study design.

2.6.NIRF Image Analysis Method: In Vivo & Ex vivo

In vivo and ex vivo near-infrared fluorescence (NIRF) imaging was performed using LI-COR Pearl® Small Animal Imager (LI-COR Biosciences, Lincoln, NE, USA) in rat hindlimbs with optimized settings in Image Studio™ Software (Version 5.2, LI-COR Biosciences, Lincoln, NE, USA). Live NIRF images were captured at 800 nm channel using 85 µm or 170 µm resolution settings. In the ex vivo imaging both orientations of the allograft, including the inner side (View 1) and outer side (View 2) were imaged and accounted for fluorescence signal quantification at 800 nm channel at resolution 85 µm and focus offset of 2 settings. To quantify the fluorescence signal, a multi-sampled region of interest (ROI) strategy was utilized across the transplanted rat hindlimb. The distinct regions of the transplanted hindlimb were sampled in multiple ROIs and the fluorescence signal for each region in the hindlimb was quantified using Image Studio (Version 5.2). Based on the biological view of transplanted limbs, multiple ROIs were captured and same area for each circled region was maintained to standardize measurements in experimental groups. The background signal was normalized and shown NIRF images are linked to compare the fluorescence signal intensity across all groups. GraphPad Prism 10 was used for analyzing the signal and performing one-way analysis of variance (ANOVA) for determining statistical significance in fluorescence signals.

2.7. $^{19}F/^{1}H$ Magnetic Resonance Imaging

MRI of ex vivo limb samples was performed on a 9.4 T 30-cm Bruker Biospec AV3HD scanner equipped with a 12-cm gradient set, a 72-mm $^{1}H/^{19}F$ dual resonance RF coil and ParaVision 6.0.1 (Bruker, Billerica, MA, USA). Anatomical axial images were collected with the following parameters: repetition time (TR)/echo time (TE) = 3000-5000/20-40 ms, RARE factor = 8, field of view (FOV) 70 × 70 mm^2, 256 ×

256 matrix, and up to 42 2-mm slices to cover the entire anatomy. Anatomical pseudo-sagittal images used a TR/TE = 3500/28 ms, FOV 100 × 100 mm^2, and 13-29 slices. For the corresponding fluorine images, the carrier frequency was set to the PCE frequency (~-92 ppm) precisely measured by a ^{19}F NMR spectrum on each sample[15]. For pseudo-sagittal images, a single slice encompassing the entire leg was collected to provide an overview of the fluorine signal distributed in the legs with NA = 32 and a 64 × 64 matrix. Axial images were collected with TR/TE = 3500/28 ms, a 64 × 64 matrix, 2 mm slice thickness, matching the anatomical images, and NA = 512. The whole-body rat image was acquired with the same parameters as the pseudo-sagittal images using a tiling technique. Fluorine images were rendered in hot-iron pseudocolor and overlaid on the grayscale anatomical images for anatomical context. Regional analysis of ^{19}F MRI signals was performed using VoxelTracker (Celsense, Inc., Pittsburgh, PA, USA). Anatomical features were identified from a rat atlas.[16]

2.8.Immunohistochemistry Method for Quantification of Macrophages

Histological sections were analyzed using a region-of-interest (ROI)–guided protocol to assess CD68+ macrophages. Scanned whole-slide images of CD68-immunostained tissue sections were acquired from HistoWiz, Inc. (Brooklyn, NY, USA). For quantitative analysis, QuPath (Version 0.6.0) software was used to manually draw a defined number of ROIs having similar areas across anatomically comparable regions to minimize bias and account for spatial heterogeneity. Using the software, an artificial neural network-based object classifier was created and used to distinguish between the CD68+ macrophages and other cells based on the stain intensity and cell morphology. The object classifier was applied to each of the ROIs and the data was expressed as the number of CD68+ macrophages per ROI area in mm^2.

2.9.^{19}F NMR of Excised Tissues

Tissues from the control limb and rejected limb were dissected and positioned in an NMR tube visible to receiving coils. The tissues were suspended in 200μL of PBS and 50μL deuterium oxide. A sealed capillary containing 0.1% (v/v) trifluoroacetic acid (TFA) was inserted into each NMR tube to be used as an internal reference standard. NMR spectrum was acquired using a 400 MHz Bruker BioSpin NMR spectrometer (Billerica, MA, USA).

2.10. Histology, Immunohistochemistry/Immunofluorescence, and Microscopy Imaging

Histology and Immunohistochemistry Studies [17-19]

Tissue Processing and Sectioning: All harvested tissues (skin, muscle, nerve, and lymph nodes from rodent VCAs) were immersion-fixed in **10% neutral-buffered formalin** for 24–48 hours at room temperature. Fixed specimens were processed using an automated tissue processor (Leica ASP300S) and embedded in **paraffin blocks** under vacuum infiltration. Sections were cut at **5 µm thickness** using a rotary microtome (Leica RM2235), mounted on charged glass slides, and air-dried overnight at 37 °C prior to staining.

Routine Histology: Routine histological stains were performed to assess general tissue morphology, inflammatory infiltration, and extracellular matrix organization:

Hematoxylin and Eosin (H&E): Standard H&E staining was used to evaluate tissue architecture, inflammatory cell density, and necrosis in both control and treated grafts. Slides were examined under brightfield microscopy for epidermal integrity, vascular congestion, and cellular infiltration.

Immunohistochemistry (IHC) and Immunofluorescence (IF)[20, 21]

IHC/IF staining was performed on an **OnCore Pro automated staining platform (Biocare Medical)** to standardize runs and minimize variability. Heat-induced epitope retrieval was performed using either citrate buffer (pH 6.0) or Tris–EDTA (pH 9.0), as appropriate for each antibody. Endogenous peroxidase activity (for chromogenic IHC) was quenched with 3% hydrogen peroxide, followed by protein blocking with 5% normal serum matched to the host species of the secondary antibody. Primary antibodies used in the current study included CD68 (macrophages, ThermoFisher, MA5-13324) with nuclear counterstaining with DAPI for IF (BioLegend, 422801).

Primary antibodies were incubated **overnight at 4 °C**, followed by species-appropriate secondary detection. For chromogenic IHC, HRP-conjugated secondary antibodies were applied (1:500, 30 min, room temperature) and visualized using DAB substrate with hematoxylin counterstaining. For immunofluorescence, highly cross-adsorbed Alexa Fluor-conjugated secondary antibodies (488/555/647; 1:500, 30 min, room temperature) were used, followed by DAPI nuclear counterstain (1 µg/mL).

To ensure staining specificity and technical quality, each run included **negative controls** (isotype-matched IgG and/or omission of primary antibody) and **positive control tissues** (spleen, lymph node, and sciatic nerve). [22, 23]

Secondary Antibodies and Detection

For chromogenic immunohistochemistry (IHC), species-appropriate, **highly cross-adsorbed horseradish peroxidase (HRP)-conjugated secondary antibodies** were used—anti-mouse IgG(H+L)-HRP and anti-rabbit IgG(H+L)-HRP (Biocare Medical or Abcam). Detection employed a **3,3′-diaminobenzidine (DAB)** substrate with hematoxylin counterstain.

For immunofluorescence (IF), **F(ab′)$_2$ fragment, highly cross-adsorbed Alexa Fluor 488 (FITC), 555 (**TRITC**), or 647** (Cy5) secondaries (anti-mouse; anti-rabbit; Invitrogen/Thermo Fisher) were applied at **1:500 dilution for 30 min at room temperature**. Nuclei were counterstained with **DAPI (1 µg/mL).**

To minimize background in rat tissues, polymer-based anti-mouse/anti-rabbit detection systems or F(ab′)$_2$-based secondaries were combined with a **rat serum block**.

Secondary antibodies and detection reagents used for chromogenic and fluorescent applications are summarized in Table S2 below:

Table S1: Secondary antibodies and detection reagents (chromogenic IHC and IF) [24-29]

Application	Antibody / Reagent	Fluor / Enzyme	Host Species	Vendor	Catalog #	Dilution / Use
IHC	Anti-Mouse IgG(H+L)-HRP	HRP	Goat	ThermoFisher / Biocare	32210 / HP6012	1:500 (30 min, RT)
IHC	Anti-Rabbit IgG(H+L)-HRP	HRP	Goat	ThermoFisher / Abcam	31460 / ab6721	1:500 (30 min, RT)
IF	Alexa Fluor 488 $F(ab')_2$ Anti-Mouse IgG(H+L)	Alexa 488	Goat	Invitrogen	A11017	1:500 (30 min, RT)
IF	Alexa Fluor 555 $F(ab')_2$ Anti-Mouse IgG(H+L)	Alexa 555	Goat	Invitrogen	A21422	1:500 (30 min, RT)
IF	Alexa Fluor 647 $F(ab')_2$ Anti-Rabbit IgG(H+L)	Alexa 647	Goat	Invitrogen	A21245	1:500 (30 min, RT)

*IHC = immunohistochemistry; IF = immunofluorescence

Integration with PCE-NE fluorescence channels (DiR)

For experiments incorporating macrophage tracking, recipient-derived PFC-NE signals were interpreted in conjunction with tissue histology/IF[30-37]. Specifically, we detected recipient-associated PCE-NE signal in the **DiR/800 nm** channel and assessed its spatial correspondence with CD68+ macrophage distributions on matched sections.[38]

Image acquisition and quantitative analysis

Brightfield and fluorescence images were acquired using an **Olympus BX63 motorized fluorescence microscope** equipped with a **DP80 dual CCD camera** and an **X-Cite 120LED Boost** illumination system. Whole-slide scanning was performed using an **Olympus SlideView VS200** digital slide scanner. Imaging settings (exposure, illumination intensity, gain) were held constant across experimental groups. Images were saved as uncompressed 16-bit TIFF files when applicable. Quantitative analysis (cell counts and/or fluorescence intensity within standardized regions of interest) was performed using **ImageJ (NIH)** and **OlyVIA (Olympus)** by observers blinded to treatment group.

3. SUPPLEMENTAL FIGURES

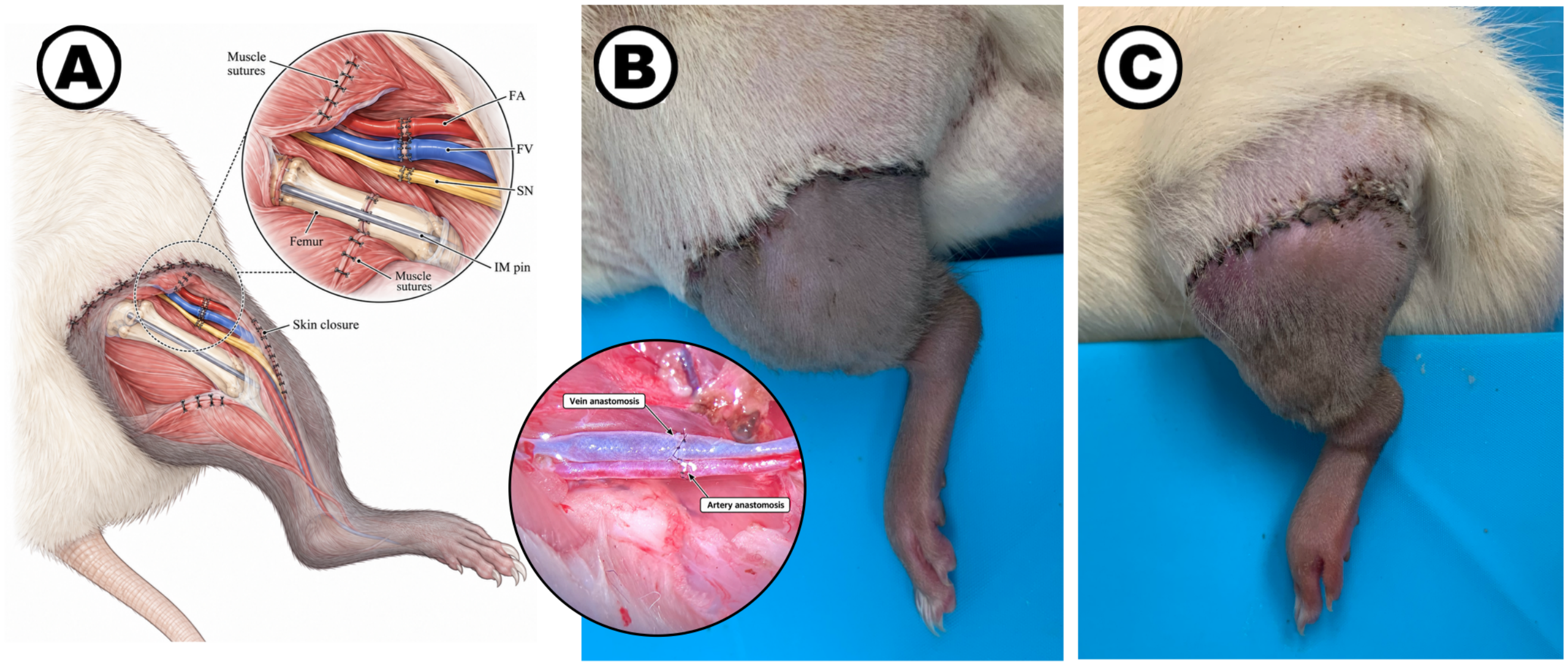

Figure S1: Panel A: Schematic illustration of the rat hindlimb vascularized composite allotransplantation model. Brown Norway (BN) donor hindlimb (dark gray) transplanted at the mid-femoral level onto a Wistar Furth (WF) recipient (white) showing femoral osteosynthesis using an intramedullary (IM) pin, microsurgical end-to-end anastomoses of the femoral artery (FA) and femoral vein (FV), sciatic nerve (SN) coaptation with epineural sutures, and muscle approximation using interrupted sutures. The magnified insets highlight the reconstructed neurovascular and musculoskeletal structures, while the external suture line delineates the donor–recipient skin junction. Panel B: Immediate post operative clinical appearance of the limb transplant. Panel C: POD 5 showing Grade II-III rejection.

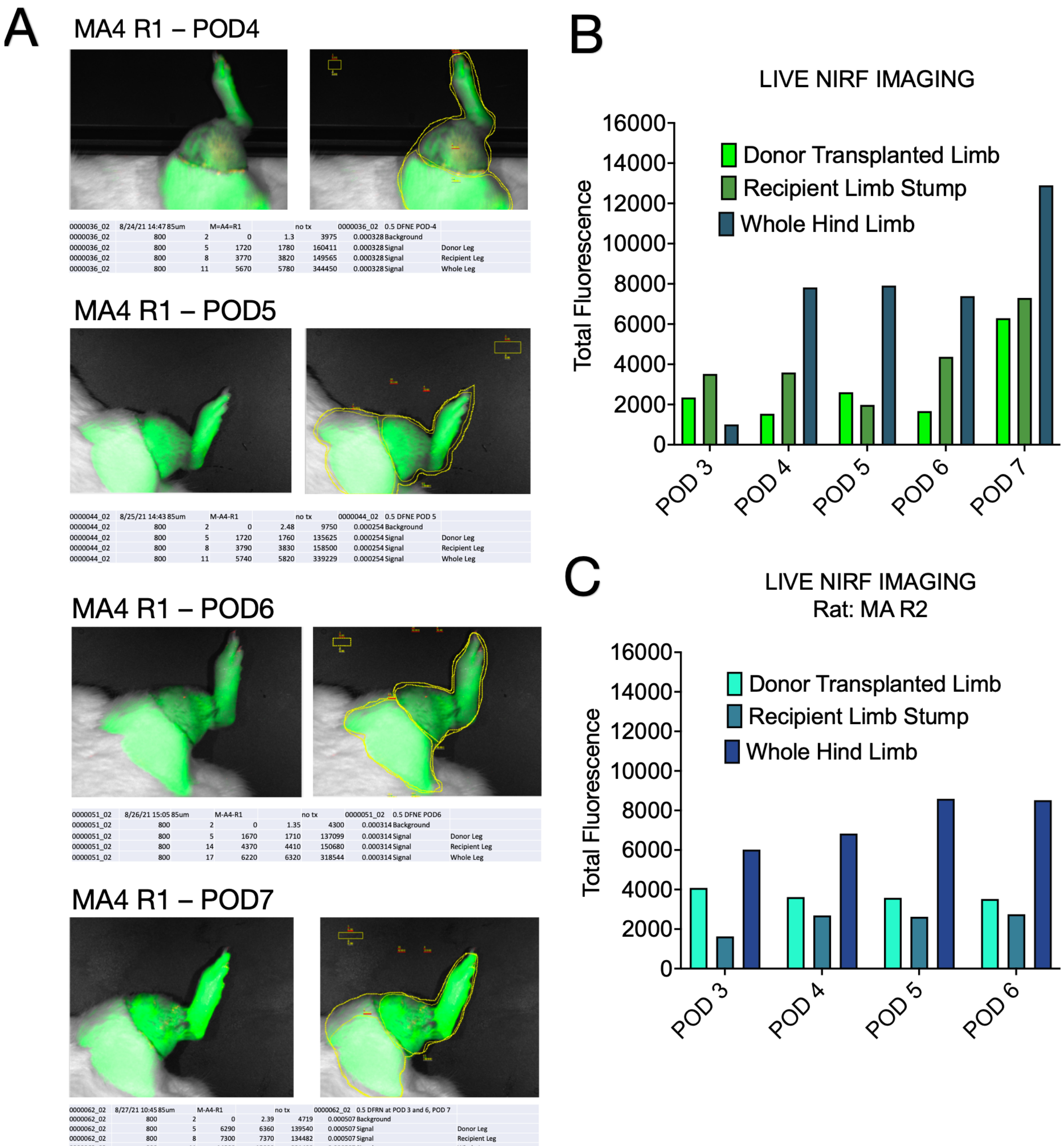

Figure S2: Longitudinal NIRF imaging of live rats undergoing acute rejection following hindlimb transplant. The images were collected from POD3-POD7, where transplanted leg is positioned into the focused view in LiCOR Pearl Trilogy imager. The images are collected at 800 nm channel. A) Series of representative sequential images with three ROIs – donor transplanted limb, recipient limb stump, where the transplanted limb was attached, and the whole hindlimb, which combines the recipient stump and the transplanted limb. B) Quantification of NIRF signal from select ROIs for each time point in a representative recipient following limballotransplantation; C) Quantification of NIRF signal following allotransplantation surgery.

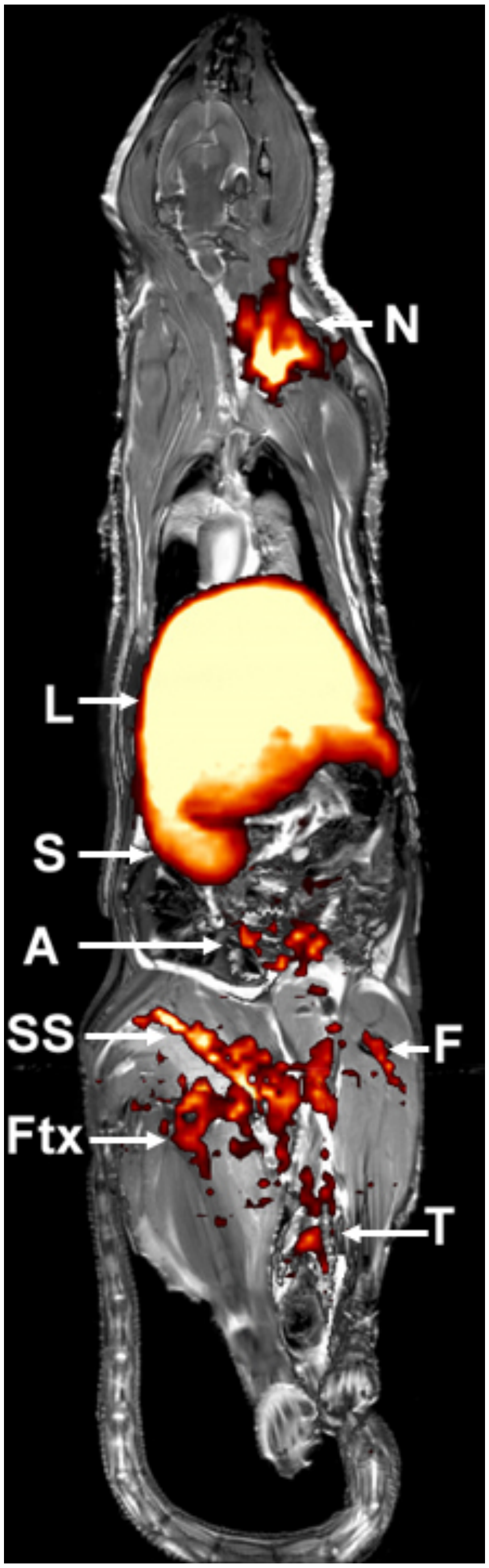

Figure S3. Whole rat MRI with an anatomical ^{1}H image (greyscale) overlaid with ^{19}F image rendered in "Hot-Iron" pseudo color. The ^{19}F image is a projection of the fluorine signal through the whole animal. Labels show specific regions in the body where PCE-NE accumulates, N is the neck area, L is the liver, S is the spleen, A is the abdomen, SS is the surgical site from the transplanted leg, F is the femur from the contralateral leg whilst Ftx is the femur from the transplanted leg and T is the testes.

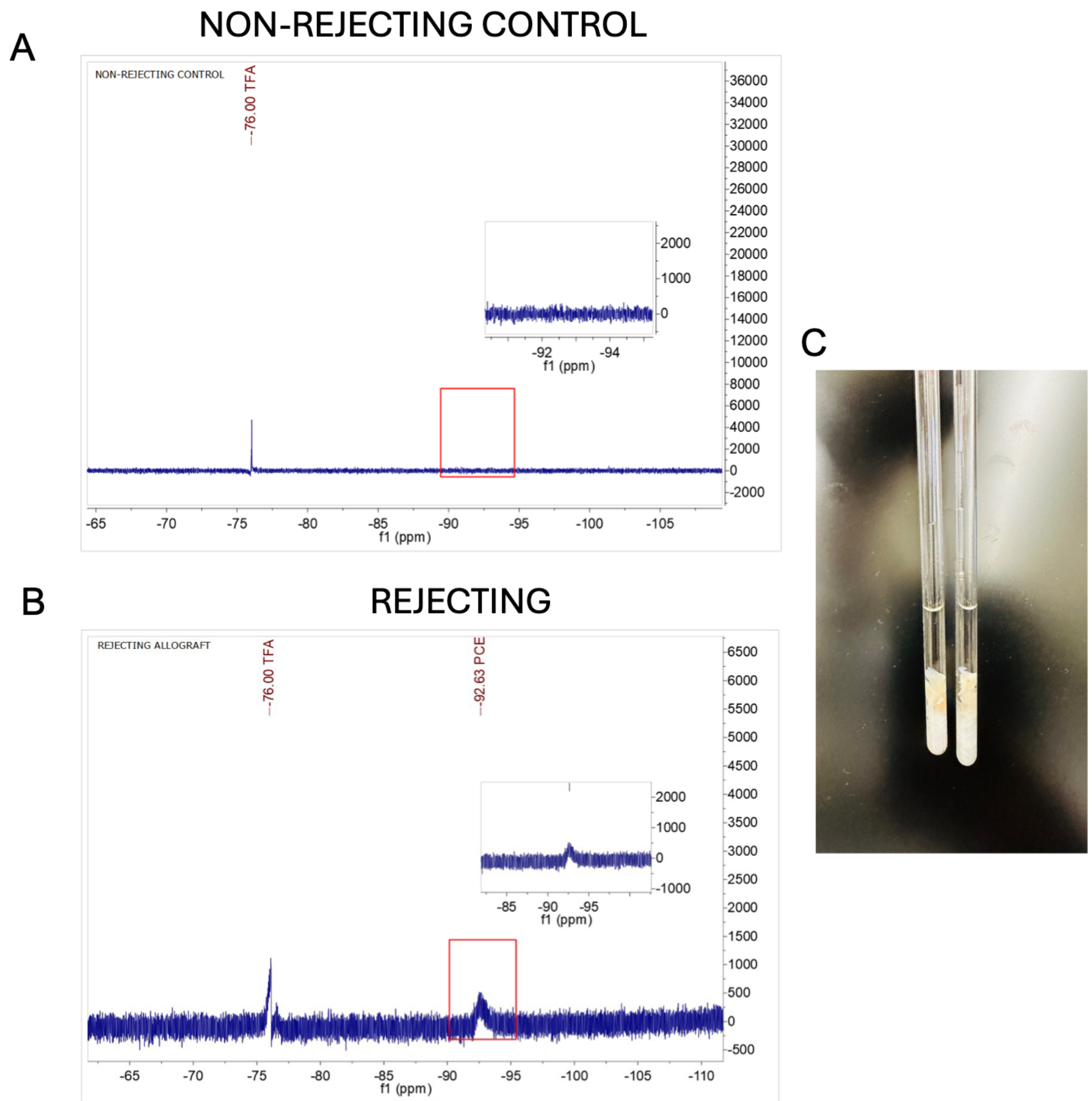

Figure S4: Representative ^{19}F NMR spectra were acquired from dissected tissues from the control/non-rejecting limb (A) and a rejected limb (B). Tissues were collected at POD6 following three doses of PCE-NE. C) Image shows the NMR tubes with collected tissues residing on top of the glass wool in a mixture of water and deuterated water next to a capillary containing 0.1% of trifluoroacetic acid (TFA) serving as a ^{19}F NMR spectral reference.

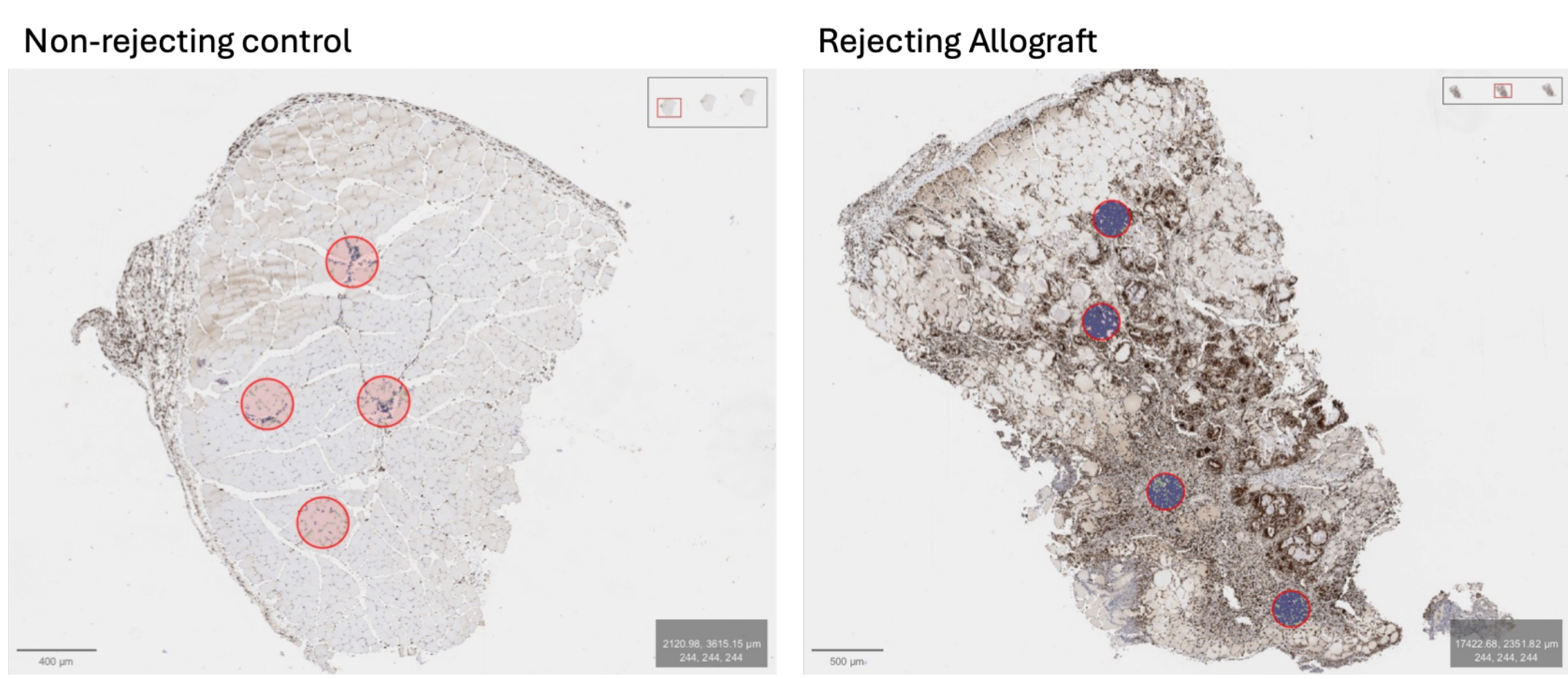

Figure S5. Representative histological image showing a randomized region of interest (ROI) selected for quantitative analysis using QuPath 0.6.0.

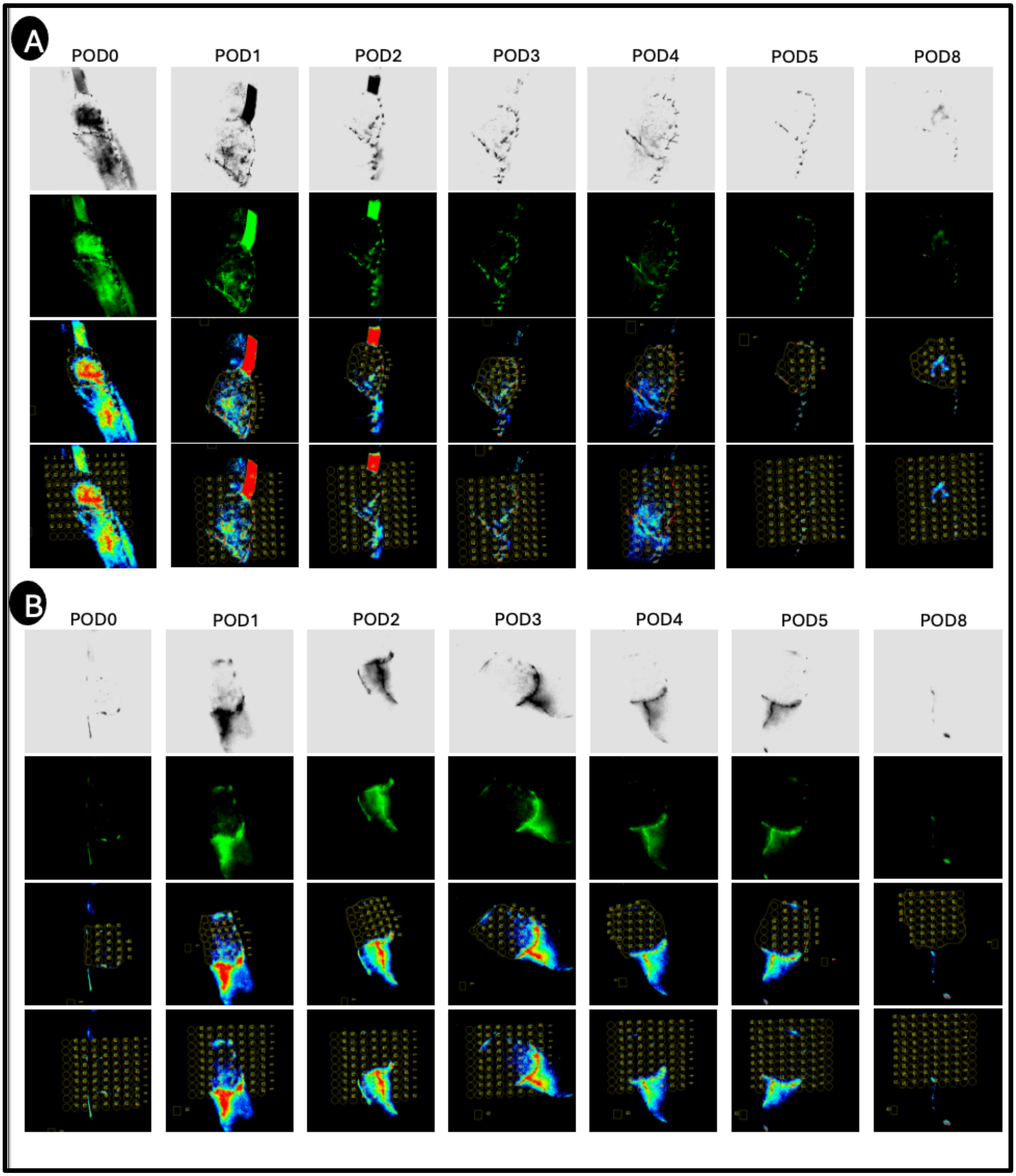

Figure S6: Near-infrared Fluorescent (NIRF) live imaging of RFF rejection model. Nonhuman primates were imaged at days 0, 1, 2, 3, 4, 5, 7 (B) and 8 (A) post skin flap surgery. Images are shown in black and white (first row), green channel (second row), Multichannel with overlayed flap regions of interest (third row), and multichannel with overlayed expanded region of interest (fourth row).

4. SUPPLEMENTAL INFORMATION REFERENCES:

1. Herneisey M, Janjic JM. Multiple linear regression predictive modeling of colloidal and fluorescence stability of theranostic perfluorocarbon nanoemulsions. Pharmaceutics. 2023; 15: 1103.
2. Vichare R, Janjic J. Perfluorocarbon nanoemulsions in drug delivery: design, development, and manufacturing. Theranostics. 2025; 15: 3013.
3. Janjic JM, Vasudeva K, Saleem M, Stevens A, Liu L, Patel S, et al. Low-dose NSAIDs reduce pain via macrophage targeted nanoemulsion delivery to neuroinflammation of the sciatic nerve in rat. Journal of neuroimmunology. 2018; 318: 72–9.
4. Gorantla VS, Prabhune KA, Perez-Abadia G, Ildstad ST, Maldonado C, Orhun HI, et al. Composite tissue allotransplantation in chimeric hosts: part I. Prevention of graft-versus-host disease. Transplantation. 2003; 75: 922–32.
5. Prabhune KA, Gorantla VS, Maldonado C, Perez-Abadia G, Barker JH, Ildstad ST. Mixed allogeneic chimerism and tolerance to composite tissue allografts. Microsurgery. 2000; 20: 441–7.
6. Prabhune KA, Gorantla VS, Perez-Abadia G, Francois CG, Vossen M, Laurentin-Perez LA, et al. Composite tissue allotransplantation in chimeric hosts part II. A clinically relevant protocol to induce tolerance in a rat model. Transplantation. 2003; 76: 1548–55.
7. Kostereva NV, Wang Y, Fletcher DR, Unadkat JV, Schnider JT, Komatsu C, et al. IGF-1 and Chondroitinase ABC Augment Nerve Regeneration after Vascularized Composite Limb Allotransplantation. PLoS One. 2016; 11: e0156149.
8. Hautz T, Zelger BG, Weissenbacher A, Zelger B, Brandacher G, Landin L, et al. Standardizing skin biopsy sampling to assess rejection in vascularized composite allotransplantation. Clin Transplant. 2013; 27: E81–90.
9. Kanitakis J. The challenge of dermatopathological diagnosis of composite tissue allograft rejection: a review. J Cutan Pathol. 2008; 35: 738–44.
10. Cendales LC, Farris AB, Rosales I, Elder D, Gamboa-Dominguez A, Gelb B, et al. Banff 2022 Vascularized Composite Allotransplantation Meeting Report: Diagnostic criteria for vascular changes. Am J Transplant. 2024; 24: 716–23.
11. Cendales LC, Kanitakis J, Schneeberger S, Burns C, Ruiz P, Landin L, et al. The Banff 2007 working classification of skin-containing composite tissue allograft pathology. Am J Transplant. 2008; 8: 1396–400.
12. Etra JW, Grzelak MJ, Fidder SAJ, Kolegraff K, Bonawitz S, Shores J, et al. A Skin Rejection Grading System for Vascularized Composite Allotransplantation in a Preclinical Large Animal Model. Transplantation. 2019; 103: 1385–91.
13. Gorantla VS, Demetris AJ. Acute and chronic rejection in upper extremity transplantation: what have we learned? Hand Clin. 2011; 27: 481–93, ix.
14. Sarhane KA, Khalifian S, Ibrahim Z, Cooney DS, Hautz T, Lee WP, et al. Diagnosing skin rejection in vascularized composite allotransplantation: advances and challenges. Clin Transplant. 2014; 28: 277–85.
15. Kadayakkara DK, Damodaran K, Hitchens TK, Bulte JW, Ahrens ET. (19)F spin-lattice relaxation of perfluoropolyethers: Dependence on temperature and magnetic field strength (7.0-14.1T). J Magn Reson. 2014; 242: 18–22.
16. Hayakawa T, Iwaki T. A color atlas of sectional anatomy of the rat: Adthree Publishing; 2008.
17. Feldman AT, Wolfe D. Tissue processing and hematoxylin and eosin staining. Methods Mol Biol. 2014; 1180: 31–43.

18. Sy J, Ang LC. Microtomy: Cutting Formalin-Fixed, Paraffin-Embedded Sections. Methods Mol Biol. 2019; 1897: 269–78.
19. Fischer AH, Jacobson KA, Rose J, Zeller R. Hematoxylin and eosin staining of tissue and cell sections. CSH Protoc. 2008; 2008: pdb prot4986.
20. Shi SR, Liu C, Taylor CR. Standardization of immunohistochemistry for formalin-fixed, paraffin-embedded tissue sections based on the antigen-retrieval technique: from experiments to hypothesis. J Histochem Cytochem. 2007; 55: 105–9.
21. Shi SR, Cote RJ, Taylor CR. Antigen retrieval immunohistochemistry: past, present, and future. J Histochem Cytochem. 1997; 45: 327–43.
22. Ramos-Vara JA, Miller MA. When tissue antigens and antibodies get along: revisiting the technical aspects of immunohistochemistry--the red, brown, and blue technique. Vet Pathol. 2014; 51: 42–87.
23. Ramos-Vara JA. Technical aspects of immunohistochemistry. Vet Pathol. 2005; 42: 405–26.
24. Buchwalow I, Samoilova V, Boecker W, Tiemann M. Non-specific binding of antibodies in immunohistochemistry: fallacies and facts. Sci Rep. 2011; 1: 28.
25. Mao S, Xiong G, Johnson BN, Cohen NA, Cohen AS. Blocking Cross-Species Secondary Binding When Performing Double Immunostaining With Mouse and Rat Primary Antibodies. Front Neurosci. 2021; 15: 579859.
26. Manning CF, Bundros AM, Trimmer JS. Benefits and pitfalls of secondary antibodies: why choosing the right secondary is of primary importance. PLoS One. 2012; 7: e38313.
27. Pina R, Santos-Diaz AI, Orta-Salazar E, Aguilar-Vazquez AR, Mantellero CA, Acosta-Galeana I, et al. Ten Approaches That Improve Immunostaining: A Review of the Latest Advances for the Optimization of Immunofluorescence. Int J Mol Sci. 2022; 23.
28. Duraiyan J, Govindarajan R, Kaliyappan K, Palanisamy M. Applications of immunohistochemistry. J Pharm Bioallied Sci. 2012; 4: S307–9.
29. Hussaini HM, Seo B, Rich AM. Immunohistochemistry and Immunofluorescence. Methods Mol Biol. 2023; 2588: 439–50.
30. Vichare R, Kulahci Y, McCallin R, Zor F, Selek FN, Liu L, et al. Theranostic nanoemulsions suppress macrophage-mediated acute inflammation in rats. J Nanobiotechnology. 2025; 23: 80.
31. Vichare R, Janjic J. Perfluorocarbon nanoemulsions in drug delivery: design, development, and manufacturing. Theranostics. 2025; 15: 3013–34.
32. Patel S, Pinky PP, Das AC, Copus JS, Aardema C, Crelli C, et al. Perfluorocarbon Nanoemulsions for Simultaneous Delivery of Oxygen and Antioxidants During Machine Perfusion Supported Organ Preservation. Pharmaceutics. 2026; 18.
33. Nichols JM, Crelli CV, Liu L, Pham HV, Janjic JM, Shepherd AJ. Tracking macrophages in diabetic neuropathy with two-color nanoemulsions for near-infrared fluorescent imaging and microscopy. J Neuroinflammation. 2021; 18: 299.
34. Janjic JM, Patel SK, Bagia C. Utilization of Near-Infrared Fluorescent Imaging for Pharmaceutically Relevant Applications. Methods Mol Biol. 2016; 1444: 97–108.
35. Janjic JM, McCallin R, Liu L, Crelli C, Das AC, Troidle A. In vitro Quality Assessments of Perfluorocarbon Nanoemulsions for Near-infrared Fluorescence Imaging of Inflammation in Preclinical Models. Bio Protoc. 2023; 13: e4842.
36. Herneisey M, Liu L, Lambert E, Schmitz N, Loftus S, Janjic JM. Development of Theranostic Perfluorocarbon Nanoemulsions as a Model Non-Opioid Pain Nanomedicine Using a Quality by Design (QbD) Approach. AAPS PharmSciTech. 2019; 20: 65.

37. Das AC, Reddy GA, Newaj SM, Patel S, Vichare R, Liu L, et al. Design of Experiments Leads to Scalable Analgesic Near-Infrared Fluorescent Coconut Nanoemulsions. Pharmaceutics. 2025; 17.
38. Janjic JM, Srinivas M, Kadayakkara DK, Ahrens ET. Self-delivering nanoemulsions for dual fluorine-19 MRI and fluorescence detection. J Am Chem Soc. 2008; 130: 2832–41.
